# Genetic deletion or pharmacological inhibition of soluble epoxide hydrolase reduces brain damage and attenuates neuroinflammation after intracerebral hemorrhage

**DOI:** 10.1186/s12974-017-1005-4

**Published:** 2017-11-25

**Authors:** Chun-Hu Wu, Song-Kun Shyue, Tai-Ho Hung, Shin Wen, Chao-Chang Lin, Che-Feng Chang, Szu-Fu Chen

**Affiliations:** 1 Graduate Institute of Life Sciences, National Defense Medical Center, Taipei, Taiwan, Republic of China; 2 Institute of Biomedical Sciences, Academia Sinica, Taipei, Taiwan, Republic of China; 3 Department of Obstetrics and Gynecology, Chang Gung Memorial Hospital, Taipei, Taiwan, Republic of China; 4 College of Medicine, Chang Gung University, Taoyuan, Taiwan, Republic of China; 5 Department of Physiology and Biophysics, National Defense Medical Center, Taipei, Taiwan, Republic of China; 6 Department of Physical Medicine and Rehabilitation, Cheng Hsin General Hospital, 45 Cheng Hsin Street, Taipei, Taiwan, Republic of China; 7 Department of Neurology, Yale University School of Medicine, New Haven, CT USA

**Keywords:** Soluble epoxide hydrolase, Intracerebral hemorrhage, Microglia/macrophages, Inflammation, AUDA

## Abstract

**Background:**

Inflammatory responses significantly contribute to neuronal damage and poor functional outcomes following intracerebral hemorrhage (ICH). Soluble epoxide hydrolase (sEH) is known to induce neuroinflammatory responses via degradation of anti-inflammatory epoxyeicosatrienoic acids (EET), and sEH is upregulated in response to brain injury. The present study investigated the involvement of sEH in ICH-induced neuroinflammation, brain damage, and functional deficits using a mouse ICH model and microglial cultures.

**Methods:**

ICH was induced by injecting collagenase in both wild-type (WT) C57BL/6 mice and sEH knockout (KO) mice. WT mice were injected intracerebroventricularly with 12-(3-adamantan-1-yl-ureido)-dodecanoic acid (AUDA), a selective sEH inhibitor, 30 min before ICH. Expression of sEH in the hemorrhagic hemisphere was examined by immunofluorescence and Western blot analysis. The effects of genetic deletion or pharmacological inhibition of sEH by AUDA on neuroinflammatory responses, EET degradation, blood-brain barrier (BBB) permeability, histological damage, and functional deficits were evaluated. The anti-inflammatory mechanism of sEH inactivation was investigated in thrombin- or hemin-stimulated cultured microglia.

**Results:**

ICH induced an increase in sEH protein levels in the hemorrhagic hemisphere from 3 h to 4 days. sEH was expressed in microglia/macrophages, astrocytes, neurons, and endothelial cells in the perihematomal region. Genetic deletion of sEH significantly attenuated microglia/macrophage activation and expression of inflammatory mediators and reduced EET degradation at 1 and 4 days post-ICH. Deletion of sEH also reduced BBB permeability, matrix metalloproteinase (MMP)-9 activity, neutrophil infiltration, and neuronal damage at 1 and 4 days. Likewise, administration of AUDA attenuated proinflammatory microglia/macrophage activation and EET degradation at 1 day post-ICH. These findings were associated with a reduction in functional deficits and brain damage for up to 28 days. AUDA also ameliorated neuronal death, BBB disruption, MMP-9 activity, and neutrophil infiltration at 1 day. However, neither gene deletion nor pharmacological inhibition of sEH altered the hemorrhage volume following ICH. In primary microglial cultures, genetic deletion or pharmacological inhibition of sEH by AUDA reduced thrombin- and hemin-induced microglial activation. Furthermore, AUDA reduced thrombin- and hemin-induced P38 MAPK and NF-κB activation in BV2 microglia cultures. Ultimately, AUDA attenuated N2A neuronal death that was induced by BV2 microglial conditioned media.

**Conclusions:**

Our results suggest that inhibition of sEH may provide a potential therapy for ICH by suppressing microglia/macrophage-mediated neuroinflammation.

**Electronic supplementary material:**

The online version of this article (10.1186/s12974-017-1005-4) contains supplementary material, which is available to authorized users.

## Background

Intracerebral hemorrhage (ICH), the spontaneous extravasation of blood into the brain parenchyma, is the second most frequent stroke subtype after ischemic stroke [1]. ICH has high mortality and morbidity; 40–50% of patients die within the first month following an ICH, and only 20% will regain functional independence [1]. ICH-induced brain damage includes initial mechanical damage due to the expanding hematoma and compression and subsequent development of inflammatory and oxidative processes that primarily result from the presence of blood components. Increasing evidence suggests that the neuroinflammatory response participates in the progression of brain injury following ICH [2] and contributes to neurological deterioration and poor outcomes in patients [3]. Reduction of the ICH-induced neuroinflammatory cascade remains a promising therapeutic target to reduce secondary brain damage and improve patient recovery [2, 4, 5]. Neuroinflammation following ICH involves activation of microglia, the brain’s resident macrophages, and recruitment of peripheral leukocytes to the perihematomal region. Activated microglia/macrophages and peripheral leukocytes secrete injurious proinflammatory factors, including cytokines, inducible nitric oxide synthase (iNOS), reactive oxygen species (ROS), and matrix metalloproteinases (MMP) [6]. These inflammatory events may induce blood-brain barrier (BBB) disruption and brain edema, which ultimately lead to neuronal death and neurological deterioration [6]. Microglia and macrophage activation involves diverse phenotypes with different physiological roles that have been historically classified as a classically proinflammatory phenotype or an alternatively restorative phenotype [7, 8]. In models of neuroinflammation, proinflammatory microglia/macrophage activation is characterized by upregulation of proinflammatory mediators (e.g., tumor necrosis factor [TNF]-α, interleukin [IL]-1β, and nitric oxide [NO]) and is associated with neurotoxicity [9]. On the other hand, restorative microglia/macrophages secrete anti-inflammatory cytokines and neurotrophic factors and are involved in wound healing and repair [9]. Considering these opposing roles of microglia/macrophages, recent studies have shown that inhibition of proinflammatory microglia/macrophage activation had beneficial effects on ICH [10, 11].

Epoxyeicosatrienoic acids (EETs), metabolites of arachidonic acid derived from the cytochrome P450 (CYP450) enzymes, are potent intracellular lipid signaling molecules that possess important biological activities, including vasodilatation, anti-inflammation, and cellular signaling regulation [12]. Recent animal studies have shown that exogenous administration of 14,15-EET is protective against experimental ischemic brain injury [13, 14], supporting a neuroprotective role for EETs in the damaged brain. However, the half-life of EETs in vivo is very short, as they are rapidly hydrolyzed by soluble epoxide hydrolase (sEH) to the less active vicinal diol compounds, dihydroxyeicosatrienoic acids (DHETs). Therefore, the sEH hydrolysis is thought to be a major determinant of EET bioavailability. Indeed, a number of animal studies have supported the hypothesis that increasing ratios of EETs to DHETs through genetic deletion or pharmacological inhibition of sEH offers protection against experimental cerebral ischemia [15, 16], parkinsonism [17], and seizures [18]. Clinically, patients with genetic polymorphisms that reduce sEH activity show improved outcomes after subarachnoid hemorrhage [19]. Importantly, EETs can have anti-inflammatory effects through suppressing NF-κB activation [20]. EETs can also reduce lipopolysaccharide (LPS)-induced proinflammatory macrophage activation and polarize macrophages toward a restorative phenotype [21]. Pharmacological inhibition or genetic deletion of sEH consequently reduces inflammation after experimental stroke [14], seizures [18], and spinal cord injury [22]. However, it remains unclear if sEH also contributes to microglia/macrophage activation and subsequent neuronal death after ICH and thus whether the inhibition of sEH might be beneficial. To address this issue, the present study aimed to examine the effects of genetic and pharmacological inhibition of sEH on ICH-induced neuroinflammation, neuronal damage, and long-term functional recovery.

## Methods

### Animals

All animal protocols were carried out according to the Guide for the Care and Use of Laboratory Animals published by the US National Institutes of Health (NIH publication no. 85-23, revised 1996) and were approved by the Animal Research Committee at Cheng Hsin General Hospital (animal permit numbers CHGH-102-01 and CHGH-103-09). Male wild-type (WT) C57BL/6 mice were obtained from BioLASCO (Taipei, Taiwan). Male sEH knockout (KO) mice in the C57BL/6 background were purchased from Jackson Laboratory (Bar Harbor, ME, USA). Animals were housed under conditions of controlled temperature (22–25 °C) and humidity (40–60%) with a 12-h/12-h dark cycle and were allowed free access to water and food.

### Cell culture

#### BV2 and neuro-2A (N2A) cell line cultures

The mouse BV2 microglial and N2A cell lines were cultured in Dulbecco’s modified Eagle’s media (DMEM; Gibco, Bethesda, MD, USA) supplemented with 10% heat-inactivated fetal bovine serum (FBS; Gibco), 100 U/mL penicillin, and 100 μg/mL streptomycin in a humidified atmosphere of 5% CO_2_ at 37 °C as previously described [5, 23].

#### Primary microglial culture

Primary mouse microglia culture was prepared from the cortices of postnatal day 7 (P7) WT or sEH KO mice as described previously [24]. Briefly, cortices were sliced and digested in 0.5 mg/mL papain for 15 min at room temperature. The cells were plated with Roswell Park Memorial Institute (RPMI) 1640 Medium (Gibco) supplemented with 10% heat-inactivated FBS, 100 U/mL penicillin, and 100 μg/mL streptomycin. After 2 weeks, the microglial cells were separated from the astrocytes by shaking at 2000 rpm for 10 min (37 °C). Non-adhered cells were eliminated, and microglial cells were replated onto 24-well plates (1 × 10^5^ cells per well) in RPMI 1640 Medium with 10% inactivated FBS and used for the experiments 24 h after. The purity of cultured microglia was higher than 99%, as verified by glial fibrillary acidic protein (GFAP) and ionized calcium-binding adaptor molecule 1 (Iba1) immunohistochemical staining (Additional file 1: Figure S1).

### Culture drug treatment

BV2 microglia and primary mouse microglia were stimulated with either 10 U/mL thrombin or 10 μM hemin in the absence or presence of varying concentrations of 12-(3-adamantan-1-yl-ureido)-dodecanoic acid (AUDA; Cayman, Ann Arbor, MI, USA) or 14,15-epoxyeicosa-5(*Z*)-enoic acid (EEZE; 1 μM, Cayman) for 3, 6, or 24 h. For collection of conditioned media, BV2 microglia were plated and incubated with 100 ng/mL LPS, 10 U/mL thrombin, or 10 μM hemin in the absence or presence of 10 μM AUDA for 24 h. Cell-free supernatant fractions were applied to N2A cells for 48 h to evaluate the changes in cell viability. All group experiments were performed independently four or five times.

### Protocol for animal experiments

Eight- to 10-week-old mice were randomized into different treatment groups using computer-generated random numbers. All outcome measurements and analyses were performed in a blinded manner. Sample sizes are determined by power analysis based on our pilot data and previous studies. A total of 409 mice (306 WT and 103 sEH KO) were used. Mice that had neurologic deficit scores greater than 15 or less than 3 at 3 h post-ICH were excluded from the study. Fifty-nine WT and 17 sEH KO mice were excluded due to neurologic deficit standard or death after ICH (WT: 59/260; sEH KO: 17/80). Sixty-five additional sham-operated control mice were used for biochemical assays and Evans blue quantification (44 WT and 21 sEH KO). Four normal mice were used for Western blot analysis (2 WT and 2 sEH KO). Four studies were conducted. The first study examined the specificity of the anti-sEH antibody and the temporal profile and cellular localization of sEH expression after ICH. These assessments included Western blot analysis (*n* = 5–6/group) and double immunofluorescence labeling. The second study investigated the anti-inflammatory and neuroprotective effects of sEH deletion. Experimental methods employed were as follows: (1) histology staining (*n* = 5–7/group), (2) cytokine enzyme-linked immunosorbent assays (ELISAs) and matrix metalloproteinase (MMP)-9 zymography (*n* = 6/group), (3) EET and 14,15-DHET ELISAs (*n* = 4/group), (4) Evans blue dye extravasation (*n* = 5–6/group), and (5) hemoglobin assay (*n* = 5/group). Cytokine ELISAs and MMP-9 analyses were performed on the same experimental group whereas other tests were performed on different experimental groups. The third study evaluated the anti-inflammatory and neuroprotective effects of sEH inhibition by AUDA, a selective sEH inhibitor, which has been widely used to evaluate the biological role of sEH [15, 18]. Experiments were as follows: (1) histology staining (*n* = 5–6/group), (2) Western blot analysis, cytokine ELISAs, and MMP-9 zymography (*n* = 6–7/group), (3) EET and 14,15-DHET ELISAs (*n* = 4/group), (4) Evans blue dye extravasation (*n* = 6/group), (5) hemoglobin assay (*n* = 5/group), and (6) behavioral and body weight assessments (*n* = 14/group). Western blot, cytokine ELISAs, and MMP-9 analyses were performed on the same experimental group.

### Intracerebral hemorrhage model

ICH model was induced by collagenase injection as previously described [25]. Mice were intraperitoneally anesthetized with sodium pentobarbital (65 mg/kg) and injected with bacterial collagenase (0.0375 U of type VII-S in 1 μL of saline; Sigma-Aldrich, St. Louis, MO, USA) into the stratum using stereotactic coordinates: 0.8 mm anterior and 2.5 mm lateral to the bregma, 2.5 mm in depth, and at a rate of 0.1 μL/min over 10 min. The needle was left in place for an additional 20 min to prevent reflux. Sham-operated mice were injected with an equal volume of normal saline in the same manner. Saline instead of heat-inactivated collagenase was infused into sham-operated brains as there were no differences in protein levels of cleaved caspase-3 (cCP-3), Iba1, and IL-1β between the saline- and heat-inactivated collagenase-injected groups (Additional file 2: Figure S2).

### Intracerebroventricular injection

AUDA (Cayman; 1 or 10 μM in 0.5 μL of 1% dimethyl sulfoxide, DMSO) or an equal volume of vehicle (1% DMSO) was given by intracerebroventricular (i.c.v.) injection 30 min before ICH as previously described [26]. Briefly, a 30-gauge needle of a Hamilton syringe was inserted into the lateral ventricle (stereotaxic coordinates: 0.5 mm posterior and 1 mm lateral to the bregma, 2 mm in depth). Then, AUDA or vehicle was infused with an infusion pump for 10 min at a rate of 0.05 μL/min. The needle was maintained in the infusion site for 20 min before removal to prevent reflux, and the ICH surgery was performed immediately thereafter.

### Histology and immunohistochemistry

Mice were sacrificed by transcardial perfusion following terminal anesthesia with sodium pentobarbital (80 mg/kg, i.p.) for histological examinations. Frozen sections (10 μm) were stained with cresyl violet, Fluoro-Jade B (FJB, a marker of degenerating neurons), and terminal deoxynucleotidyl transferase-mediated dUTP-biotin nick end labeling (TUNEL; In situ Cell Death Detection Kit, Roche Molecular Biochemicals) as previously described [5]. For immunostaining, sections were incubated with primary antibodies of rabbit anti-Iba1 (a microglia/macrophage marker; 1:1000; Wako Pure Chemical Industries, Osaka, Japan) or rabbit anti-myeloperoxidase (MPO, a neutrophil marker; 1:1000; Dako, Carpinteria, CA, USA) overnight [5]. Further colorimetric detection was carried out using diaminobenzidine as the peroxidase substrate. The specificity of the staining reaction was assessed by omission of the primary antibody and substitution of the primary antibody with non-immune rabbit serum.

### Double immunofluorescence staining

To assess the cellular source of sEH, double immunofluorescence labeling was performed by simultaneous incubation of sections with rabbit anti-sEH (1:1000; Santa Cruz Biotechnology, Santa Cruz, CA, USA) overnight at 4 °C with mouse anti-CD11b (a microglia/macrophage marker; 1:100; Abcam, Cambridge, UK), rat anti-GFAP (an astrocyte marker; 1:200; Invitrogen, Camarillo, CA, USA), mouse anti-neuronal nuclei antigen (NeuN, a neuronal marker; 1:100, Millipore, Billerica, MA, USA), and rat anti-CD31 (an endothelial cell marker; 1:100; BD Biosciences, San Jose, CA, USA). To assess proinflammatory microglia/macrophages, sections were incubated overnight at 4 °C with rabbit anti-Iba1 (1:1000; Wako), together with rat anti-CD16/32 (a classic M1 activation marker; 1:100; BD Biosciences). Sections were washed, then incubated with Alexa Fluor 488- or Alexa Fluor 594-conjugated secondary antibodies (1:500; Molecular Probes, Eugene, OR, USA) for 2 h.

### Quantification of Iba1, MPO, FJB, and TUNEL staining

FJB, TUNEL, MPO, or Iba1 staining was quantified in three consecutive sections from the hemorrhagic core at the level of 0.24 mm from the bregma. The number of positive cells was counted in an area of 920 × 860 μm^2^ in 10–12 non-overlapping fields immediately adjacent to the hematoma using a magnification of ×200 as previously described [5]. Iba1-positive resting microglia/macrophages were defined as resting if they contained relatively small cell bodies (< 7.5 μm in diameter) with long slender processes [27]. Microglia/macrophages were defined as activated when a cell body increased in size compared to resting microglia with short, thick processes and intense immunointensity. Activated microglia/macrophages were defined based on a combination of morphological criteria and a cell body diameter cutoff of 7.5 μm. FJB-, MPO-, and Iba1-positive cells were expressed as cells/field. Quantification of TUNEL staining was expressed as (TUNEL-stained nuclei/DAPI-stained nuclei) × 100%. Analyses were performed by two experimenters who were blinded to all animal groups. Inter-rater reliability was within 10%.

### Injury volume and hemispheric enlargement assessment

Injury volumes, striatum atrophy, and striatum enlargement ratios were quantified using cresyl violet-stained sections at 20 rostral-caudal levels that were spaced 200 μm apart as previously described [5]. Sections were analyzed using ImageJ software (version 1.50i; National Institutes of Health, Bethesda, MD, USA). The volume measurement was computed by summation of the areas multiplied by the interslice distance (200 μm). Striatum atrophy and striatum enlargement to account for brain edema were assessed using the following formula: ([ipsilateral striatum volume − contralateral striatum volume] / contralateral striatum volume) × 100%. Analyses were performed by two experimenters who were blinded to all animal groups. Inter-rater reliability was within 10%.

### Immunocolocalization analysis and quantification

Colocalization of the microglia/macrophage marker (Iba1) and proinflammatory microglia/macrophage activation marker (CD16/32) was analyzed as previously described [28, 29]. Briefly, colocalization signals (in yellow) in merged images were obtained from the microglia/macrophage marker (in red) and the proinflammatory activation marker (in green) and turned into the gray pixel map by ImageJ software. The degree of colocalization was expressed in arbitrary units.

### ELISA

IL-1β, IL-6, macrophage inflammatory protein-2 (MIP-2), and monocyte chemoattractant protein-1 (MCP-1) (R&D Systems, Minneapolis, MN, USA), along with EETs (MyBiosource, San Diego, CA, USA), and 14,15-DHET (Detroit R&D Inc., Detroit, MI, USA), were measured in brain homogenates or cell lysates using commercially available ELISA kits.

### Blood-brain barrier permeability

Evans blue dye (2% in normal saline, 4 mL/kg) was injected into the tail vein and allowed to circulate for 1 h. The mice were then transcardially perfused with phosphate-buffered saline (PBS), and the ipsilateral hemispheric samples were homogenized in 1 mL of 60% trichloroacetic acid by sonication. After centrifugation at 4500 rpm for 15 min at 4 °C, the supernatants were diluted with ethanol (1:4). The absorbance of Evans blue in the supernatant was measured at 620 nm. Evans blue concentrations were calculated and expressed as μg/g brain tissue using a standard curve.

### Gelatin zymography

Zymography was performed as previously described [25]. Briefly, equal amounts of protein were loaded and separated on a 10% Tris-glycine gel with 0.1% gelatin as the substrate. Then, the gel was washed and renatured with 2.5% Triton X-100 buffer. After incubation with developing buffer at 37 °C for 24 h, the gel was stained with 0.05% Coomassie R-250 dye (Sigma-Aldrich) for 30 min and destained. Gelatinolytic activity (MMP-9: ~ 97 kDa; MMP-2: ~ 72 kDa) was determined as clear bands at the appropriate molecular weights.

### Hemoglobin assay

The hemoglobin contents of ICH brains were quantified using a spectrophotometric assay according to previously described methods [5]. Distilled water (300 μL) was added to the hemorrhagic hemisphere, followed by homogenization for 30 s and sonication on ice for 1 min. After centrifugation at 13,000 rpm for 30 min, 20 μL of supernatant was reacted with Drabkin reagent (80 μL; Sigma-Aldrich) for 15 min. Optical density was then measured at a wavelength of 545 nm to assess the concentration of cyanmethemoglobin. The hemorrhage volume can then be calculated from the linear relation between the optical density and hemoglobin concentration.

### Western blot analysis

Western blot analyses were performed as previously described [30]. Protein samples obtained from tissue or BV2 microglia homogenates were separated on 8–12% sodium dodecyl sulfate-polyacrylamide gels, transferred to Immobilon-P membranes (Millipore), and probed overnight at 4 °C with primary antibodies including rabbit anti-sEH (1:1000) and rabbit anti-P65 (1:1000) from Santa Cruz; rabbit anti-iNOS (1:1000) and rabbit anti-cyclooxygenase (COX-2 1:1000) from Cayman; rabbit anti-cCP-3 (1:1000), rabbit anti-p-P38 (1:1000), rabbit anti-P38 (1:2000), rabbit anti-p-C-Jun N-terminal kinase (JNK, Thr183/Tyr185, 1:1000), rabbit anti-JNK (1:2000), rabbit anti-p-extracellular signal-regulated kinase p44/42 (ERK p44/42; Thr202/Tyr204, 1:1000), rabbit anti-ERK (1:2000), and rabbit anti-p-P65(1:1000) from Cell Signaling (Danvers, MA, USA); and mouse anti-β-actin (1:10,000, Sigma-Aldrich). Protein band intensities were quantified using ImageJ software and were normalized to the corresponding β-actin intensity.

### Behavioral testing

All the behavioral tests were performed before ICH and at 1, 4, 7, 14, 21, and 28 days after ICH. Mice were pretrained for both rotarod and beam walking tests for 3 days.

#### Modified neurological severity score (mNSS)

The mNSS provides an index of motor, sensory, reflex, and balance tests [31]. The neurological function of each animal was graded on a scale of 0–18, and one point was given for the inability to perform each test or for the absence of a testing reflex.

#### Rotarod test

The rotarod test was used to measure motor function and balance [32]. Briefly, the speed of an accelerating rotarod was increased from 6 to 42 rpm over 7 min, and the running time was recorded until mice fell off.

#### Beam walking test

Motor function and coordination were also assessed by measuring the ability of mice to pass across an elevated beam [32]. The latency time for a mouse to traverse the beam (not to exceed 60 s) and the hindlimb performance as it crossed the beam (based on a 1 to 7 rating scale) were recorded. The hindlimb scoring system rates the inability to balance on the beam as a score of “1” and the ability to traverse the beam normally with both paws on the beam surface and fewer than two foot slips as a “7.” For the rotarod and beam walking tests, three measurements per trial were recorded 1 h before ICH (baseline) and at 1, 4, 7, 14, 21, and 28 days post-ICH.

#### Elevated body swing test

The body swing test for evaluating asymmetrical motor behavior was conducted as previously described with slight modifications [33]. Mice were suspended vertically by the tail, inverted approximately 1 in. from the floor. A swing was counted when the animal moved its head > 10 degrees away from the vertical axis to either side. The frequency and direction of the swing behavior were recorded for 30 s. ICH mice exhibited significantly biased swing activity toward the direction contralateral to the hemorrhagic side. The total number of swings made to the left side was divided by the total number of swings to obtain the percentages of the left bias of the swings.

### NO production and cell viability

NO production was assessed by measuring the nitrite levels of the culture supernatants using the Griess reagent (Sigma-Aldrich). The nitrite content in the samples was calculated based on a standard curve prepared with known concentrations of sodium nitrite. Cell viability was assessed using 3-(4,5-dimethyl-2-thiazolyl)-2,5-diphenyl-2*H*-tetrazolium bromide (MTT) assay (Sigma-Aldrich). Data are presented as the percentage of control group. The experiments were repeated four to five times using different batches of primary cultures.

### Statistical analyses

Data are presented as the mean and standard error of the mean (mean ± S.E.M.). One-way or two-way analysis of variance (ANOVA) followed by a post hoc Bonferroni test was used for multiple groups to determine significant differences. Student’s *t* test was used to test the difference between two groups. Statistical significance was set at *P* < 0.05.

## Results

### Increased sEH protein expression in mice after ICH

**Fig. 1:**
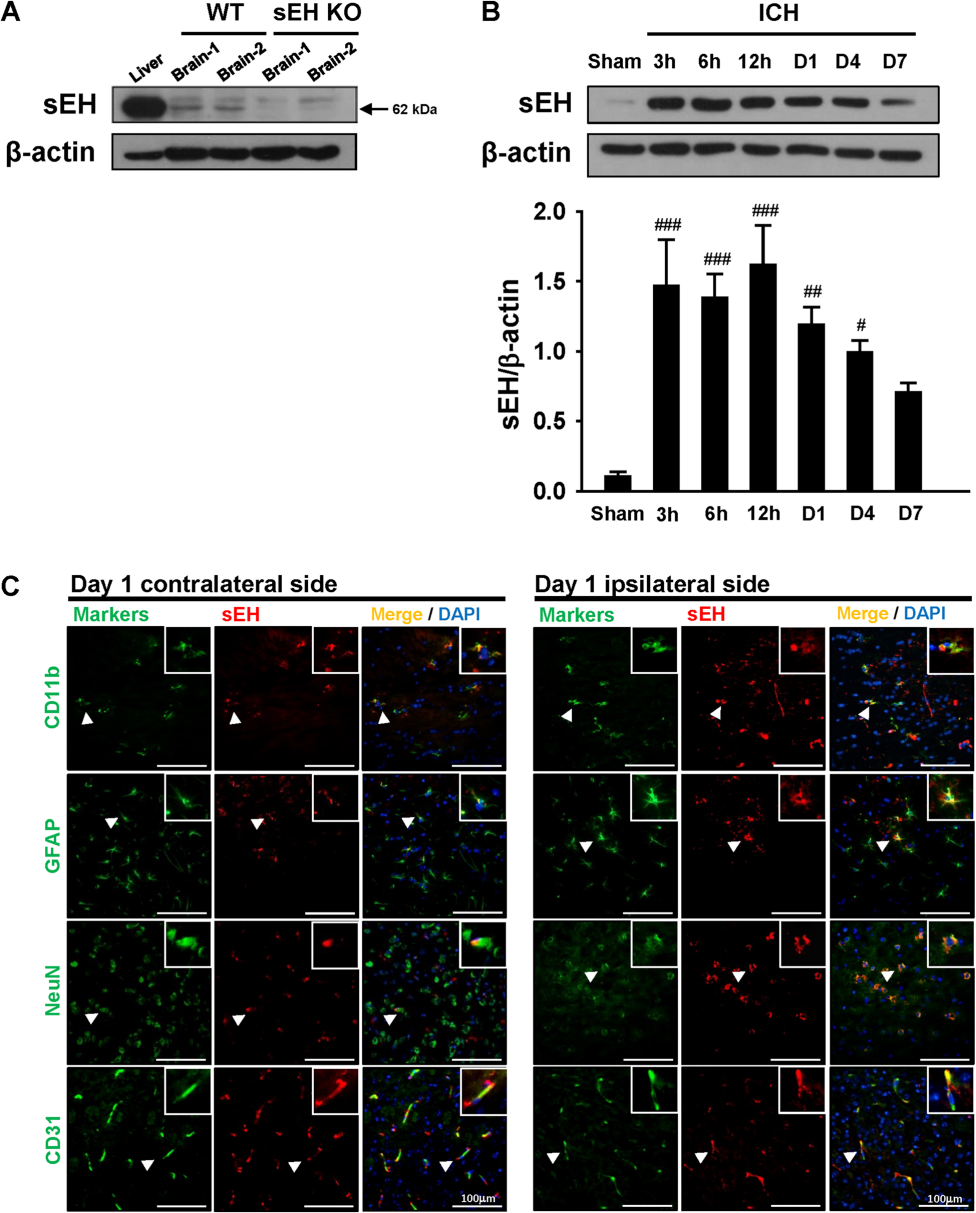
Temporal profiles and cellular distribution of sEH in WT mouse brains subjected to ICH. **a** Immunoblots of sEH protein in normal brains and the liver tissue. **b** Representative immunoblots and quantitative data of sEH protein levels in the hemorrhagic hemispheres from ICH or sham-operated mice. Values are mean ± S.E.M.; ^#^ *P* < 0.05, ^##^ *P* < 0.01, and ^###^ *P* < 0.001 vs. sham group (*n* = 5–6 mice/group, one-way ANOVA). **c** Double-labeling shows colocalization of sEH protein with microglia (CD11b), astrocytes (GFAP), neurons (NeuN), and endothelial cells (CD31) in the perihematomal area of both the contralateral and ipsilateral hemispheres at 1 day post-ICH. White arrowheads indicate colocalization. The inset images represent higher magnification of the colocalization regions in the corresponding images. Sections were stained with DAPI (blue) to show all nuclei. The scale bar is 100 μm

We first examined whether sEH protein expression was altered in brain tissues following collagenase-induced ICH. Western blot results showed that sEH was expressed in sham-operated brains and the liver tissue (as a positive control) of WT mice, but absent in sEH KO brains (Fig. 1a). The sEH protein level in the hemorrhagic hemisphere was significantly increased compared with the sham control by 3 h (*P* < 0.001) following ICH and remained elevated at 6 h (*P* < 0.001), 12 h (*P* < 0.001), 1 day (*P* = 0.003), and 4 days (*P* = 0.026, Fig. 1b). The sEH protein level was decreased at 7 days. We next defined the cell types that expressed sEH at 1 day post-ICH. Dual-label immunofluorescence demonstrated that sEH was expressed in microglia/macrophages, astrocytes, neurons, and endothelial cells both in the perihematomal region of the striatum and in the contralateral hemisphere (Fig. 1c).

### Deletion of sEH reduced microglia/macrophage activation and expression of proinflammatory cytokines and chemokines and EET degradation after ICH

**Fig. 2:**
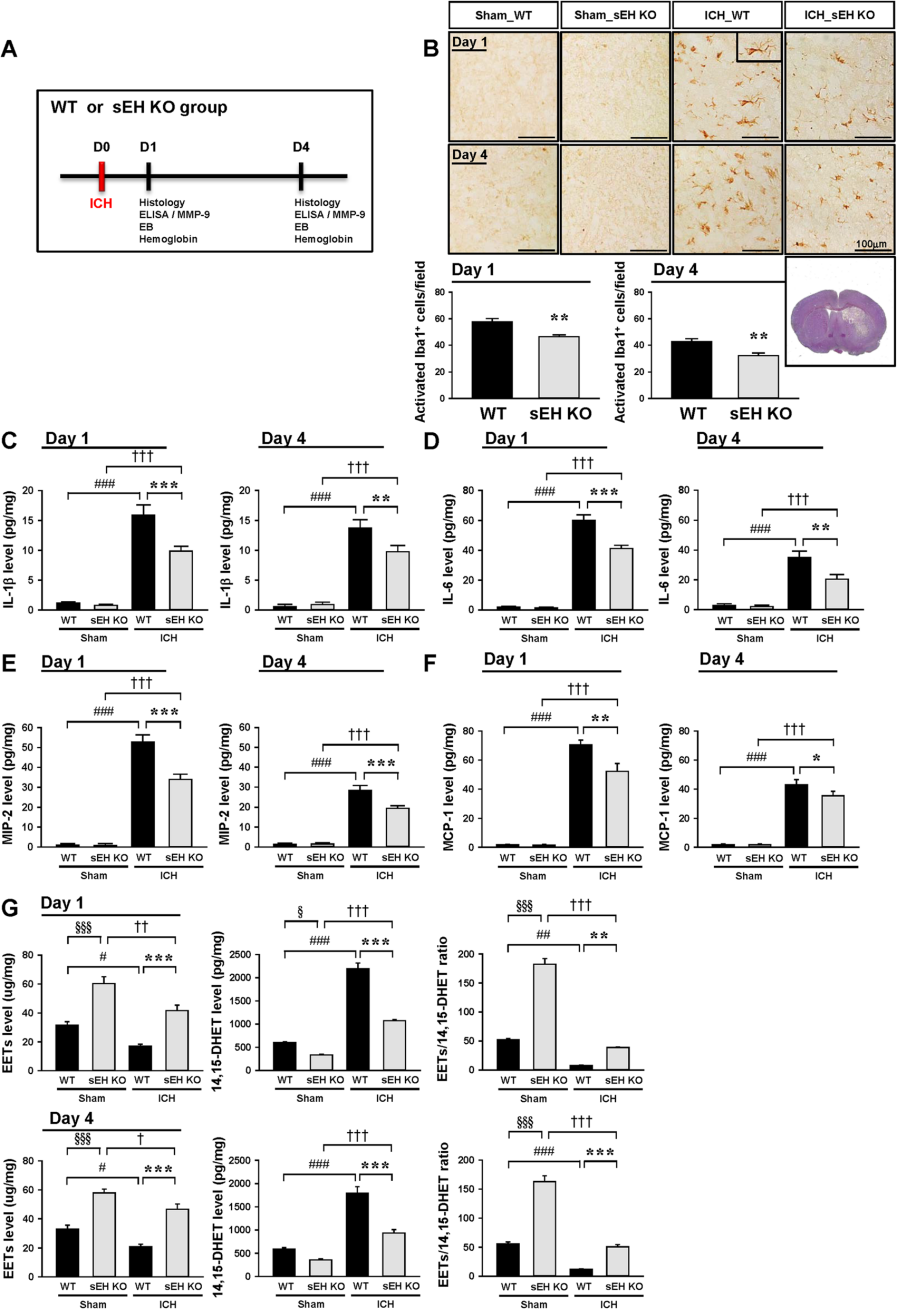
Deletion of sEH reduced microglia/macrophage activation and expression of proinflammatory mediators and EET degradation after ICH. **a** The experimental design scheme used to study the effect of sEH deletion. ELISA enzyme-linked immunosorbent assay, EB Evans blue. **b** Iba1 staining and quantitative data at 1 and 4 days post-ICH. The inset is a representative activated microglia/macrophage at higher magnification. Low magnification of a cresyl violet-stained brain section of a core hemorrhagic region at 0.24 mm from the bregma (bottom right). The white box indicates the location of representative images. The number of Iba1-positive cells is expressed as the mean number per field of view (0.8 mm^2^). The scale bar is 100 μm. Bar graphs of **c** IL-1β, **d** IL-6, **e** MIP-2, **f** MCP-1 protein levels, and **g** EET protein level, EET/14,15-DHET ratio, and 14,15-DHET protein level, as assessed by ELISA at 1 and 4 days post-ICH. Values are mean ± S.E.M.; ^#, †^ *P* < 0.05, ^##, ††^ *P* < 0.01, and ^###, †††^ *P* < 0.001 vs. sham group; *^, §^ *P* < 0.05, **P < 0.01, and ***^, §§§^ *P* < 0.001 vs. WT group (*n* = 6 mice/group for Iba1 staining, Student’s *t* test; *n* = 4–6 mice/group for ELISA, repeated measures two-way ANOVA)

Since we demonstrated that sEH was upregulated in the hemorrhagic hemisphere following ICH and was colocalized with microglia/macrophages, we hypothesized that sEH inhibition reduces neuroinflammatory responses after ICH. To test this hypothesis, we first used a loss-of-function strategy to evaluate the effect of sEH deletion on microglia/macrophage activation at 1 and 4 days post-ICH. These two time points were chosen because the inflammatory-related signals peak within 4 days after collagenase-induced ICH and decline thereafter [5, 6, 34]. At 1 and 4 days post-ICH, activated Iba1-positive microglia/macrophages accumulated at the hematoma border; however, deletion of sEH significantly reduced the number of activated Iba1-positive microglia/macrophages at 1 day (*P* = 0.0038, Fig. 2b) and 4 days (*P* = 0.0049, Fig. 2b). Deletion of sEH also significantly reduced the expression of proinflammatory mediators IL-1β, IL-6, MIP-2, and MCP-1 at 1 day (IL-1β, IL-6, and MIP-2: *P* < 0.001; MCP-1: *P* = 0.001; Fig. 2c–f) and 4 days (IL-1β, *P* = 0.008; IL-6: *P* = 0.002; MIP-2: *P* < 0.001; MCP-1: *P* = 0.038; Fig. 2c–f). As for the direct metabolic consequences of sEH deficiency, ICH caused reductions in EET levels and the EET/14,15-DHET ratio and an increase in 14,15-DHET levels in both WT and sEH KO mice at both 1 and 4 days post-ICH (all *P* < 0.05; Fig. 2g). Deletion of sEH caused a significant elevation of EET levels and the EET/14,15-DHET ratio and a decrease of 14,15-DHET levels at both time points tested post-ICH (all *P* < 0.01; Fig. 2g). Taken together, these data demonstrate that sEH deficiency mitigates the proinflammatory response after ICH.

### Deletion of sEH reduced BBB permeability, MMP-9 activity, and neutrophil infiltration in mice after ICH

**Fig. 3:**
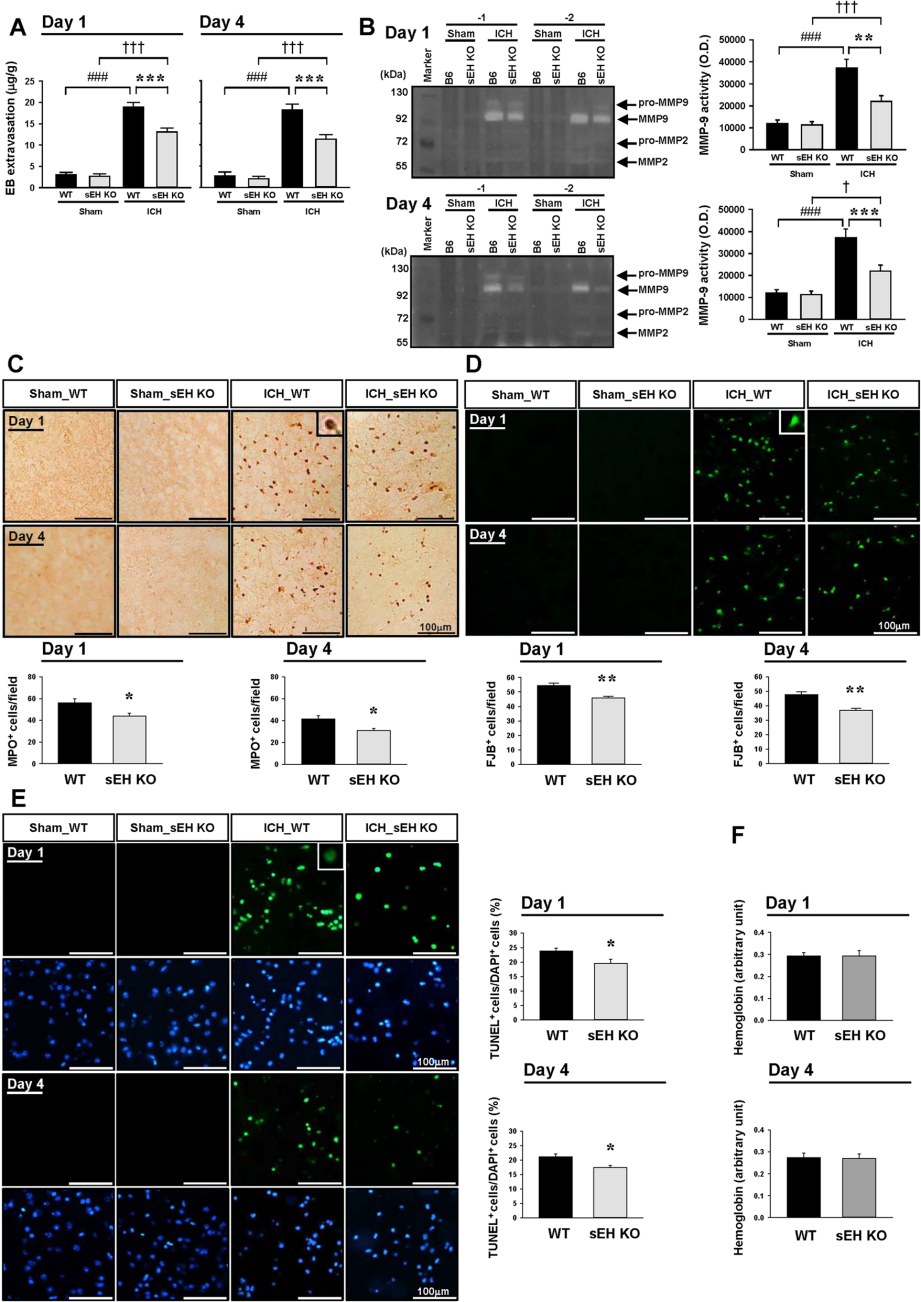
Deletion of sEH reduced BBB permeability, MMP-9 activity, neutrophil infiltration, neuronal damage, and apoptosis after ICH. **a** Quantitative analysis of Evans blue dye leakage at 1 and 4 days post-ICH. **b** Representative gelatin zymography and quantitative analysis of MMP-9 activity at 1 and 4 days post-ICH. **c** MPO, **d** FJB, and **e** TUNEL (green) stainings and quantitative data from the perihematomal area (see the white box in the cresyl violet-stained image in Fig. 2b) at 1 and 4 days. The inset image is a representative MPO-positive, FJB-positive, or TUNEL-positive cell at higher magnification. The number of MPO-positive and FJB-positive cells is expressed as the mean number per field of view (0.8 mm^2^). The percentage of TUNEL-positive cells is expressed as the percentage of TUNEL-positive nuclei over total number of DAPI-stained nuclei. Sections were stained with DAPI (blue) to show all nuclei. The scale bar is 100 μm. **f** Hemoglobin levels at 1 and 4 days. Values are mean ± S.E.M.; ^†^ *P* < 0.05, ^###, †††^ *P* < 0.001 vs. sham group; *P < 0.05, **P < 0.01, and ***P < 0.001 vs. WT group (*n* = 5–6 mice/group for Evans blue quantification and *n* = 6 mice/group for zymography, repeated measures two-way ANOVA; *n* = 6–7 mice/group for MPO and FJB staining, Student’s *t* test; *n* = 5 mice/group for TUNEL staining and hemoglobin assay, Student’s *t* test)

We next evaluated the effect of sEH deletion on BBB integrity, as BBB disruption is a major consequence of post-ICH inflammation and contributes to brain edema and subsequent neuronal damage [35]. The Evans blue content in the hemorrhagic hemispheres of the WT group was significantly attenuated by sEH deletion at both 1 and 4 days (both *P <* 0.001; Fig. 3a). To explore the possible mechanisms underlying the protective effects of BBB integrity, we also evaluated MMP-9 activity, as it disrupts the BBB by degrading the tight junction proteins and basal lamina proteins [35]. MMP-9 activity in hemorrhagic hemispheres was significantly increased in WT and sEH KO mice at both 1 and 4 days post-ICH (all *P <* 0.05; Fig. 3b), with only a slight enhancement in the sham-injured brains. Deletion of sEH significantly suppressed the induction of MMP-9 activity at both 1 day (*P* = 0.009; Fig. 3b) and 4 days post-ICH (*P <* 0.001; Fig. 3b). Additionally, ICH induces a robust infiltration of neutrophils, which exacerbate BBB breakdown and brain damage by release of cytotoxic mediators or by increasing inflammatory cell recruitment. Deletion of sEH significantly reduced neutrophil accumulation compared with WT mice in the perihematomal area at both 1 day (*P* = 0.0298; Fig. 3c) and 4 days (*P* = 0.0167). These data indicate that sEH deficiency sealed the barrier and reduced MMP-9 activity and neutrophil accumulation.

### Deletion of sEH attenuated neuronal death and apoptosis but did not alter hemorrhage volumes in mice after ICH

To determine whether the observed changes in inflammatory responses and BBB breakdown were reflected in a reduction of neuronal death and apoptosis, histological outcomes were evaluated. The number of FJB-positive degenerative neurons around the hematoma were significantly reduced in sEH KO brains compared to WT brains at both 1 day (*P* = 0.0024; Fig. 3d) and 4 days (*P* = 0.0012; Fig. 3d) post-ICH. Deletion of sEH also significantly attenuated the percentage of TUNEL-positive cells compared with WT mice at both 1 day (*P* = 0.0359; Fig. 3e) and 4 days (*P* = 0.0214; Fig. 3e). However, no significant between-group differences were found in brain hemoglobin content, an indicator of hemorrhage size at either 1 or 4 days post-ICH (Fig. 3f). This indicates that the protective mechanisms of sEH deficiency against ICH were independent of hemorrhage volumes.

### Pharmacological inhibition of sEH by the sEH inhibitor, AUDA, reduced pro-inflammatory microglia/macrophage activation and EET degradation after ICH

**Fig. 4:**
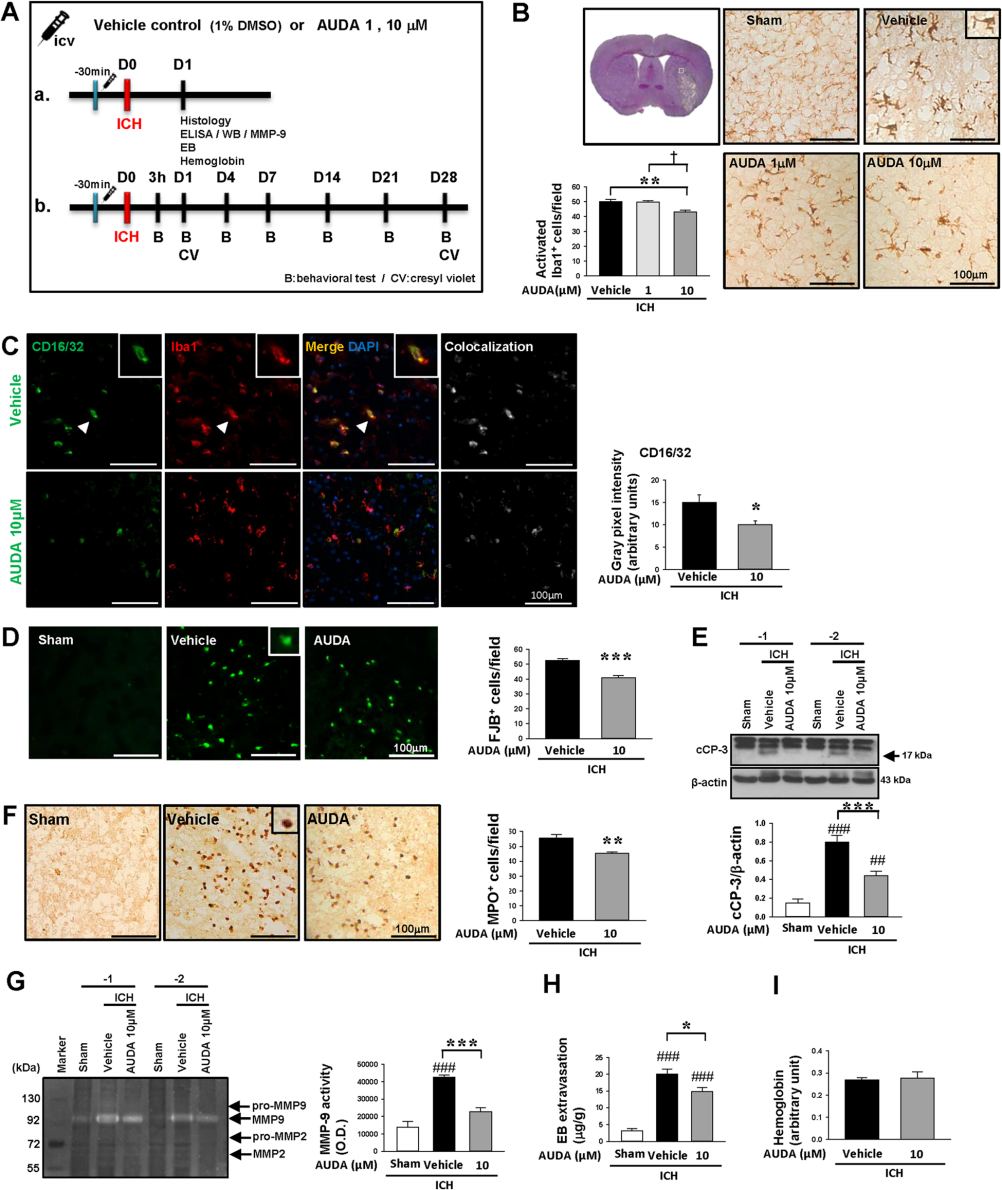
Pharmacological inhibition of sEH reduced proinflammatory microglia/macrophage activation, neuronal damage, and MMP-9 activity and attenuated BBB permeability after ICH. **a** The experimental design scheme used to study the effect of sEH inhibition by AUDA. ELISA enzyme-linked immunosorbent assay, EB Evans blue, MMP-9 matrix metalloproteinase-9, WB Western blot. **b** Iba1 staining and quantitative data at 1 day post-ICH. The inset is a representative activated microglia/macrophage at higher magnification. Low magnification of a cresyl violet-stained brain section of a core hemorrhagic region at 0.24 mm from the bregma (top left). The white box indicates the location of representative images. **c** Double-labeling shows colocalization of CD16/32 (classic activation marker) and Iba1 in the perihematomal area at 1 day. Arrowheads indicate the cell of colocalization. The inset images represent higher magnification of the colocalized regions in the corresponding images. Nuclei were stained with DAPI (blue). The bar graph shows the degree of Iba1 and CD16/32 colocalization in gray pixel intensity. **d** FJB staining and quantitative data at 1 day. The inset is a representative FJB-positive cell at higher magnification. **e** Western blot analysis of c-CP and **f** MPO staining and quantitative data at 1 day. The inset is a representative MPO-positive cell at higher magnification. **g** Representative gelatin zymography and quantitative data of MMP-9 activity and **f** hemoglobin levels at 1 day. **h** Quantification of brain Evans blue leakage at 1 day. **i** Quantification of hemoglobin contents at 1 day. The white box in the cresyl violet-stained image in **b** indicates the location of representative images for Iba1, FJB, and MPO stainings. The number of Iba1-, FJB-, and MPO-positive cells is expressed as the mean number per field of view (0.8 mm^2^). Values are mean ± S.E.M.; ^##^ *P* < 0.01, and ^###^ *P* < 0.001 vs. sham group; *P < 0.05, **P < 0.01, and ***P < 0.01 vs. vehicle group; ^†^ *P* < 0.05 vs. 1 μM AUDA group (*n* = 5 mice/group for Iba1, FJB staining, and hemoglobin assay and *n* = 6 mice/group for MPO staining, Student’s *t* test; *n* = 5 mice/group for CD16/32 and Iba1 double staining, *n* = 6 mice/group for Western blot and Evans blue quantification, and *n* = 7 mice/group for zymography, one-way ANOVA)

To explore whether the pharmacological inhibition of sEH also suppressed ICH-mediated neuroinflammation, we treated WT mice with i.c.v. injection of the sEH inhibitor AUDA (1 and 10 μM) 30 min before collagenase-induced ICH (Fig. 4a). We chose the route of i.c.v. administration to focus on the cerebral sEH inhibition effects; as sEH is found in many other organs, systemic administration of the inhibitor could have caused unexpected physiologic results in non-cerebral and vascular systems [36]. We evaluated the effect of AUDA treatment on inflammatory responses at day 1 because both microglia/macrophage activation and proinflammatory chemokine/cytokine expression peaked at this time point and declined at day 4, as shown in Fig. 2. The number of activated Iba1^+^ microglia/macrophages around the hematoma was significantly reduced following 10 μM AUDA treatment (*P* = 0.007, Fig. 4b) but not 1 μM treatment (*P* > 0.05, Fig. 4b). Levels of proinflammatory cytokines IL-1β, IL-6, and MIP-2 were significantly attenuated in the 10 μM AUDA-treated hemorrhagic brains compared with the vehicle group at 1 day post-ICH (IL-1β: *P* = 0.003; IL-6: *P* = 0.003; MIP-2: *P* = 0.008; Additional file 3: Figure S3A). However, only the IL-6 level was significantly reduced following 1 μM AUDA treatment (*P* = 0.035; Additional file 3: Figure S3A). Since 10 μM AUDA provided enhanced protection against microglial activation, the dosage of 10 μM was employed in subsequent studies. We next investigated whether AUDA affected the activation of proinflammatory microglia/macrophages, using double-immunofluorescent staining with the microglia/macrophage marker Iba1 and with the marker of proinflammatory microglia/macrophages (CD16/32). While no CD16/32-positive microglia/macrophages were observed in sham-operated brains, ICH induced an increase of CD16/32-positive microglia/macrophages at the hematoma border at 1 day post-ICH (Fig. 4c). The CD16/32-Iba1 double-positive signal around the hematoma was significantly decreased following 10 μM AUDA treatment (*P* = 0.0353; Fig. 4c). Moreover, 10 μM AUDA treatment resulted in a significantly elevated level of EET expression (*P* = 0.001), decreased EET/14,15-DHET ratio (*P* = 0.009), and reduced 14,15-DHET level (*P* = 0.006; Additional file 3: Figure S3B). These findings indicate that pharmacological inhibition of sEH using AUDA resulted in a reduction of EET degradation, accompanied by inhibition of proinflammatory microglia/macrophage activation following ICH.

### Pharmacological inhibition of sEH by AUDA reduced neuronal damage, BBB permeability, neutrophil infiltration, and MMP-9 enzymatic activity after ICH

We next evaluate whether sEH inhibition by AUDA attenuated neurodegeneration and apoptosis at the acute stage. Treatment with 10 μM AUDA significantly reduced the number of FJB-positive degenerative neurons around the hematoma and levels of cCP-3, a critical effector in apoptosis, compared with vehicle-treated mice at 1 day post-ICH (both *P* < 0.001; Fig. 4d, e). Also, AUDA administration resulted in significantly reduced neutrophil accumulation (*P* = 0.0030; Fig. 4f) and MMP-9 activity (*P* < 0.001; Fig. 4g) and attenuated levels of extracted Evans Blue, an indicator of BBB breakdown (*P* = 0.02; Fig. 4h), at 1 day post-ICH. However, no significant between-group differences were found in brain hemoglobin content at 1 day (Fig. 4i).

### Pharmacological inhibition of sEH by AUDA reduced brain tissue damage and improved long-term neurological function after ICH

**Fig. 5:**
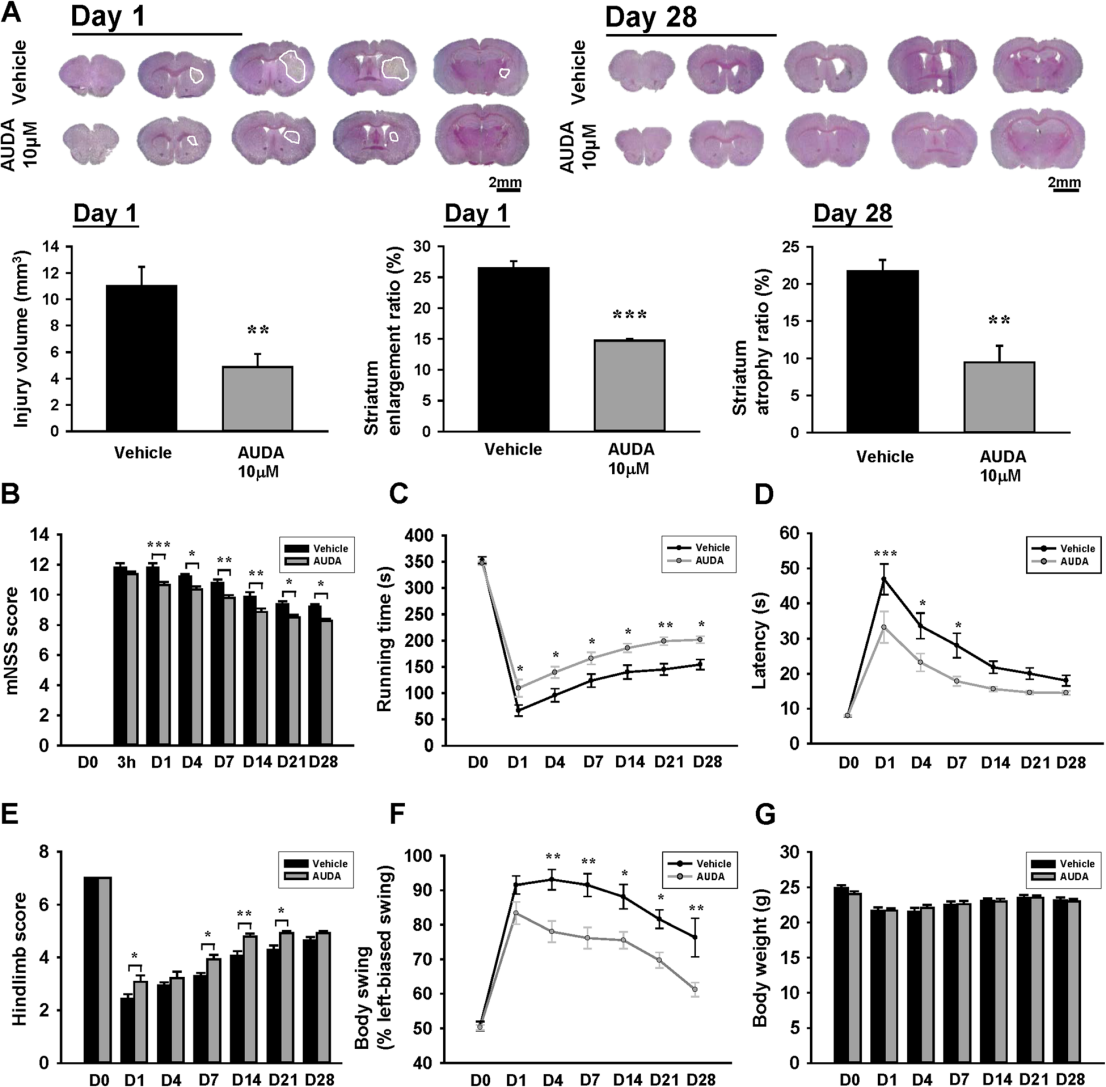
Pharmacological inhibition of sEH by AUDA reduced brain tissue damage and improved long-term neurological function after ICH. **a** Representative cresyl violet-stained images and quantitative data of lesion volumes at 1 and 28 days post-ICH. The scale bar is 2 mm. Effects of 10 μM AUDA on **b** mNSS, **c** rotarod performance, **d** beam walk traversing time, **e** hindlimb performance, **f** left-biased swings, and **g** body weight from 1 to 28 days post-ICH. Values are mean ± S.E.M.; *P < 0.05, **P < 0.01, and ***P < 0.001 vs. vehicle group (*n* = 5–6 mice/group for cresyl violet staining, Student’s *t* test; *n* = 13–14 mice/group for behavioral tests and body weight changes, repeated measures two-way ANOVA)

We further determined whether the suppression of neuroinflammation with AUDA resulted in a reduction in brain tissue damage. Treatment with 10 μM AUDA significantly reduced hemorrhagic injury volumes to 44% of the vehicle group (*P* = 0.0059; Fig. 5a) at 1 day. Striatal enlargement, an indicator of brain edema, was also significantly smaller in 10 μM AUDA-treated mice than in vehicle-treated mice (*P* < 0.001; Fig. 5a) at 1 day. At the chronic stage of ICH, 10 μM AUDA significantly reduced the degree of striatal atrophy compared with the vehicle group (*P* = 0.0012; Fig. 5a) at 28 days. We next assessed the long-term consequences of sEH inhibition on neurological recovery. At 3 h after injury, there was no difference in global neurological deficit as evaluated by mNSS between vehicle-treated and 10 μM AUDA-treated groups, indicating that injury severity was initially similar regardless of treatment (Fig. 5b). Significant improvement in neurological function was observed from 1 to 28 days in the AUDA-treated group compared with the vehicle group (all *P* < 0.05; Fig. 5b). Treatment with AUDA also significantly increased the rotarod running time from 1 to 28 days post-ICH compared with vehicle-treated mice (all *P* < 0.05; Fig. 5c). Similarly, AUDA-treated mice exhibited better beam walk performances with significantly reduced latency to cross the beam from 1 to 7 days post-ICH (*P* < 0.001 on day 1; *P* < 0.05 on days 4 and 7; Fig. 5d). The hindlimb scores were also significantly higher for AUDA-treated mice than for vehicle-treated mice at 1, 7, 14, and 21 days (all *P* < 0.05; Fig. 5e). Furthermore, the biased swing percentage was decreased in AUDA-treated mice from 4 to 28 days post-ICH compared with those that received vehicle only (all *P* < 0.05; Fig. 5f). However, there was no significant difference in body weight changes between the two groups during the 28-day observation period (all *P* > 0.05; Fig. 5g). Thus, these data demonstrate that when sEH was targeted using the selective inhibitor AUDA, EET degradation was reduced. This resulted in a reduction in proinflammatory microglia/macrophage activation and attenuation of ICH-induced neurological deficits and brain damage.

### Genetic deletion or pharmacological inhibition of sEH attenuated thrombin- and hemin-induced microglial activation and proinflammatory responses in cultured microglia

**Fig. 6:**
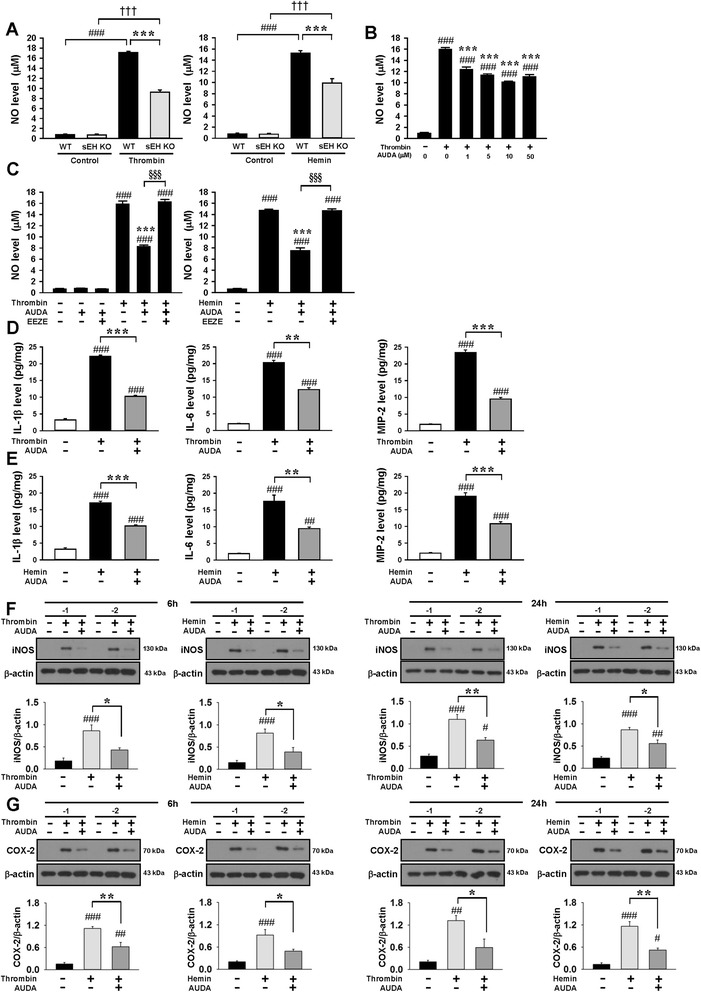
Genetic deletion or pharmacological inhibition of sEH attenuated thrombin- and hemin-induced microglial activation and proinflammatory responses in cultured microglia. **a** Effects of sEH deletion on thrombin- or hemin-induced release of NO at 24 h from the supernatant of primary microglial cultures and **b** BV2 microglial cultures. **c** Effects of AUDA and pan-EET receptor antagonist 14,15-EEZE (1 μM) on thrombin- or hemin-induced release of NO at 24 h from the supernatants of primary microglial cultures. Effects of AUDA on **d** thrombin- or **e** hemin-induced release of IL-1β, IL-6, and MIP-2 at 24 h from the supernatant of primary mouse microglial cultures. Representative immunoblots and quantitative data of **f** iNOS and **g** COX-2 protein levels following co-treatment of 10 μM AUDA with thrombin or hemin at 6 and 24 h in BV2 microglia. Values are mean ± S.E.M.; ^#^ *P* < 0.05, ^##^ *P* < 0.01, and ^###, †††^ *P* < 0.001 vs. control group; *P < 0.05, **P < 0.01, and ***P < 0.001 vs. thrombin- or hemin-stimulation group; ^†††^ *P* < 0.001 vs. thrombin- or hemin-stimulation + AUDA group (*n* = 4 experiments/group for NO levels, *n* = 3 experiments/group for ELISA, and *n* = 4–5 mice/group for Western blot, one-way ANOVA, or repeated measures two-way ANOVA)

The in vivo results demonstrated that both genetic deletion and pharmacological inhibition of sEH preserved neuronal function and reduced microglia/macrophage activation after ICH. To directly measure the effects of sEH inhibition on microglia, we next determined whether sEH modulated microglia-mediated neuroinflammation in vitro. We used thrombin, a serine protease activated during coagulation that is present at high levels during ICH, and hemin, a cytotoxic breakdown product of hemoglobin following ICH, to activate microglia. We first assessed whether the lack of sEH would affect microglial activation by comparing thrombin- and hemin-induced NO production using primary microglia derived from WT and sEH KO mice. Deletion of sEH significantly reduced 10 U/mL thrombin-induced NO production to 54% and 10 μM hemin-induced NO production to 65% of that observed for the WT group (both *P* < 0.001; Fig. 6a) at 24 h post-stimulation, suggesting that sEH deletion suppressed thrombin- and hemin-stimulated microglial activation. Next, we evaluated the effects of pharmacological inhibition by AUDA on microglial activation by using BV2 microglial cells. There was a significant increase in NO release in the culture supernatant 24 h after exposure to thrombin in BV2 microglial cells compared with unstimulated cells, but co-treatment with 1, 5, 10, or 50 μM AUDA for 24 h significantly reduced the thrombin-induced NO production to 77, 71, 63, and 69% of that observed for the vehicle control group, respectively, and 10 μM AUDA provided the highest degree of anti-inflammatory protection (all *P* < 0.001; Fig. 6b). Therefore, a dosage of 10 μM was employed for subsequent biochemical studies. We further used primary mouse microglia to confirm the results obtained from BV2 microglial cells. Like the results in BV2 cells, co-treatment with 10 μM AUDA for 24 h significantly reduced thrombin-induced NO production to 52% and hemin-induced NO production to 51% of that observed for the vehicle control group (both *P* < 0.001; Fig. 6c). To confirm whether the protective effect of the sEH inhibition was attributed to a decrease in EET metabolism, thrombin- or hemin-stimulated primary microglia were treated with 10 μM AUDA in the presence or absence of the putative pan-EET receptor antagonist 14,15-EEZE (1 μM). Antagonist 14,15-EEZE did not affect the baseline NO level, but the protective effect of AUDA on microglial activation was completely obliterated by administration of 14,15-EEZE in both thrombin- and hemin-stimulated primary microglia (Fig. 6c), indicating that the beneficial effect observed with sEH inhibition was specifically due to EETs. Treatment with 10 μM AUDA also significantly attenuated thrombin- and hemin-induced production of IL**-**1β, IL-6, and MIP-2 (all *P* < 0.01, Fig. 6d, e) in the culture medium of primary microglia. We further assessed the effect of AUDA on the two inducible enzymes, iNOS and COX2, that are expressed in activated microglia [37]. Treatment with 10 μM AUDA significantly attenuated iNOS protein expression in thrombin-stimulated BV2 cells at 6 h (50% of vehicle level, *P =* 0.015) and 24 h (58% of vehicle level, *P =* 0.003) and in hemin-stimulated BV2 cells at 6 h (48% of vehicle level, *P =* 0.011) and 24 h (64% of vehicle level, *P =* 0.01), respectively (Fig. 6f). Similarly, protein levels of COX2 were significantly reduced following AUDA treatment in thrombin-stimulated BV2 cells at 6 h (56% of vehicle level, *P =* 0.004) and 24 h (45% of vehicle level, *P =* 0.029) and in hemin-stimulated BV2 cells at 6 h (53% of vehicle level, *P =* 0.023) and 24 h (45% of vehicle level, *P =* 0.001), respectively (Fig. 6g).

**Fig. 7:**
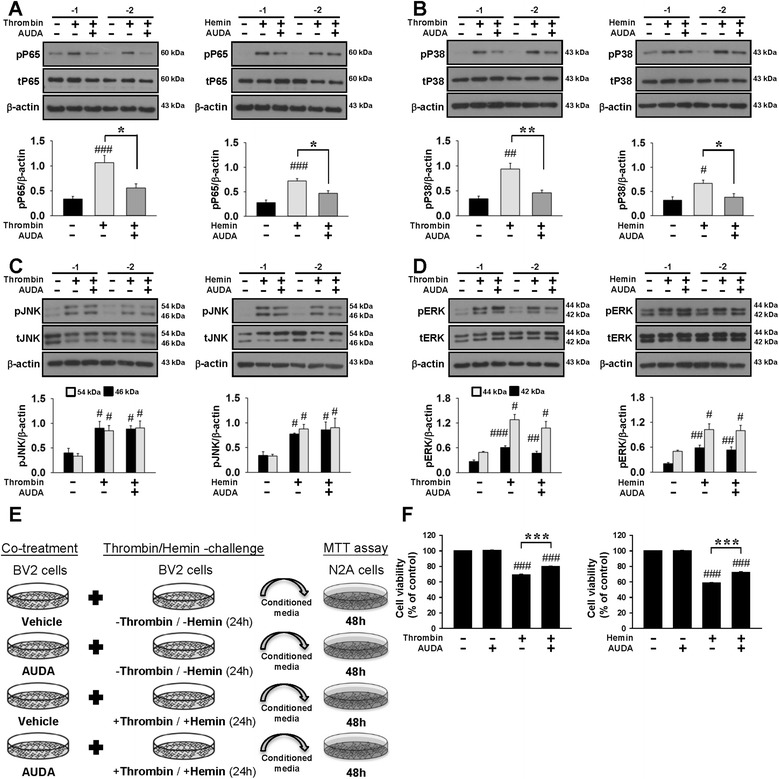
Pharmacological inhibition of sEH reduced P38 MAPK and P65 NF-κB activation in cultured microglia and attenuated microglia-mediated neurotoxicity in vitro. Representative immunoblots and quantitative data of **a** P65, **b** P38, **c** JNK, and **d** ERK phosphorylation following co-treatment of 10 μM AUDA with thrombin or hemin in BV2 microglia. **e** Experimental scheme of neuronal cell death in N2A cells in response to thrombin- or hemin-treated BV2-conditioned media with or without 10 μM AUDA pretreatment. BV2 microglia were incubated with thrombin or hemin in the absence or presence of 10 μM AUDA for 24 h. N2A cells were treated for 48 h with cell-free supernatant fractions derived from BV2 microglia and cell viability was evaluated. **f** Neuronal cell viability as assessed by the MTT assay after exposure to thrombin- or hemin-treated conditioned microglial media. Values are mean ± S.E.M.; ^#^ *P* < 0.05, ^##^ *P* < 0.01, and ^###^ *P* < 0.001 vs. control group; *P < 0.05, **P < 0.01, and ***P < 0.001 vs. thrombin- or hemin-stimulation group (*n* = 4–5 experiments/group for Western blot, *n* = 4 experiments/group for cell viability, one-way ANOVA)

We next investigated the effects of AUDA on the activation of the NF-κB, which is an inducible transcription factor that plays a crucial role in inflammatory and immune responses and can be activated by mitogen-activated protein kinases (MAPKs), a family of serine/threonine protein kinases that mediates microglial activation [38]. Co-treatment with 10 μM AUDA significantly attenuated the increased levels of pP65 Ser536, an indicator of NF-κB activation, induced by thrombin (52% of vehicle level, *P =* 0.011) and hemin (65% of vehicle level, *P =* 0.016; both in Fig. 7a). We further studied the possible effects of AUDA on the phosphorylation of P38, JNK, and ERK, the major MAPK pathway subfamilies. Co-treatment with 10 μM AUDA significantly reduced phosphorylation of P38 induced by thrombin (49% of vehicle level, *P =* 0.007) and hemin (57% of vehicle level, *P =* 0.041) without affecting the level of total P38 (Fig. 7b). However, JNK and ERK phosphorylation was not affected by AUDA treatment (all *P >* 0.05; Fig. 7c, d). Together, these results suggest that AUDA inhibits thrombin- and hemin-induced microglial activation and limits the production of inflammatory mediators by suppressing the P38 MAPK and NF-κB pathways.

### Pharmacological inhibition of sEH increased neuronal viability treated with conditioned medium from thrombin- or hemin-stimulated cultured microglia

Since our in vivo results showed that deletion and inhibition of sEH reduced microglial/macrophage activation and attenuated neuronal damage, we further hypothesized that inhibition of sEH reduced microglia-derived neurotoxic factors and thus ameliorated neuronal injury. We first assessed the direct effect of thrombin or hemin on cell viability in N2A cultures. While treatment with 10 U/mL thrombin had no direct effect on neuronal viability assessed by the MTT assay, 10 μM hemin significantly reduced viability of N2A cells to 69.2 ± 1.0% of the control level (*P* < 0.001, Additional file 4: Figure S4). Co-treatment with 10 μM AUDA significantly increased neuronal viability to 75.0 ± 1.0% compared with the vehicle control group (*P =* 0.002, Additional file 4: Figure S4). In order to mimic clinical settings, we next evaluated the effect of AUDA-treated BV2 microglia-conditioned medium on N2A cell survival in vitro. Microglia cell cultures were treated with 0.05% DMSO (control), 10 μM AUDA, thrombin or hemin, thrombin/hemin and 10 μM AUDA for 24 h (Fig. 7e). Treatment of N2A cells with conditioned medium harvested from the thrombin- or hemin-stimulated BV2 microglia at 48 h markedly decreased cell viability of N2A cells to 69.2 ± 0.6% (*P <* 0.001) or 58.6 ± 0.6% (*P <* 0.001) of the control level, respectively (Fig. 7f). When compared with N2A cells treated with conditioned media from thrombin- or hemin-stimulated BV2 microglia, cell viabilities of those treated with 10 μM AUDA were increased significantly to 79.7 ± 0.5% (*P <* 0.001) and 72.1 ± 0.9% (*P <* 0.001), respectively. These results show that the neurotoxic effects of condition media from the thrombin- or hemin-stimulated BV2 microglia were reduced by AUDA treatment, indicating that the protective effect of AUDA on neuronal viability is at least partly conferred through the inhibition of microglial-induced injury.

## Discussion

In this study, we have explored the involvement of sEH in neuroinflammatory responses and brain damage progression after ICH. Our results demonstrate that ICH increased cerebral sEH expression and that sEH was localized in microglia/macrophages. Importantly, both genetic deletion and pharmacological inhibition of sEH reduced inflammatory responses, neuronal death, and BBB permeability in mice subjected to ICH. Pharmacological inhibition of sEH also ameliorated long-term neurological deficits and brain tissue damage including acute hemorrhagic injury volume and chronic brain tissue loss after ICH. Mechanistically, AUDA attenuated thrombin- and hemin-stimulated NO and cytokine production in cultured microglia, which was associated with reduced activation of P38 MAPK and NF-κB signaling. Moreover, AUDA attenuated neuronal cell death induced by microglial conditioned media in vitro. These findings suggest an involvement of sEH in ICH-induced brain injury and subsequent alterations of EET metabolism by sEH and neuroinflammatory responses.

We observed that ICH induced an increase of cerebral sEH protein expression starting from 3 h and persisting up to 4 days. The change in sEH protein expression was mirrored in a significant decrease in cerebral EET level and elevated cerebral DHET levels. Experimentally, a number of studies have established the upregulation of sEH in various brain damage models including cerebral ischemia [14], epilepsy [18], chronic depression [39], and parkinsonism [17]. However, another study reported that brain sEH mRNA expression was significantly lower in stroke-prone spontaneous hypertensive rats than in stroke-resistant hypertensive rats, due to sequence variation in the promoter region of the gene [40]. This indicates that sEH expression in the brain is modulated by disease pathogenesis. The regulatory points for increased sEH protein expression following ICH remain undetermined. It could be attributed to ICH-induced activation of several transcriptional factors as previous studies have reported that the sEH promoter region contains multiple transcription factor binding sites including NF-κB and AP-1 [41], both of which are involved in inflammation and activated following ICH [42, 43]. Indeed, activation of NF-κB was observed as early as 15 min following rodent ICH, and this could contribute to the early upregulation of sEH [42]. Additionally, the sEH gene promoter region contains recognition sites for specificity protein (SP)-1, a transcription factor that responds to inflammatory signals and oxidative stress [44]. SP1 expression has been reported to be upregulated following experimental ischemic stroke [45] and can be regulated by NF-κB [46]; thus, it is possible that upregulation of SP1 contributes to the elevation in sEH expression. Elucidation of the mechanism(s) regarding this increased sEH expression requires further investigation.

Although previous studies have demonstrated that blocking sEH activity suppressed neuroinflammatory responses in animal models of cerebral ischemia [14], subarachnoid hemorrhage [47], epilepsy [18], and cardiac arrest [48], it is unclear whether sEH targets microglia. We demonstrated that sEH was colocalized with microglia/macrophages following ICH. Furthermore, by using primary cultured and BV2 microglia, sEH inhibition exerted a direct effect on microglial activation. As a proof of concept, thrombin and hemin (the oxidized form of heme) were used to directly induce microglial activation. Both thrombin and hemin are rapidly released following ICH and are powerful activators of microglia via the PAR-1 [49] or TLR4 receptor [50], respectively. We observed that pharmacological inhibition of sEH by AUDA or genetic deletion of sEH suppressed thrombin- and hemin-induced production of inflammatory mediators in cultured microglia. Along with the fact that AUDA attenuated neuronal cell death induced by microglial conditioned media, we provide evidence that sEH inhibition-mediated protection of damaged neurons was mediated by inhibition of the proinflammatory factors derived from activated microglia. Regarding the underlying molecular mechanisms, we found a marked reduction in the activation of NF-κB and P38 MAPK by AUDA in thrombin- and hemin-stimulated microglia without altering pERK or pJNK. The P38-MAPK pathway has been shown to play an important role in the intracellular signal transduction pathway for the production of inflammatory mediators in activated microglia [49, 51, 52]. These studies suggest that the P38 kinase is involved in several inflammatory processes, and are a potential therapeutic target for treatment of brain damage such as ICH [49, 52] or traumatic brain injury [51]. We and others have also reported that inhibition of P38 kinase with pharmacological approaches reduced ICH-induced inflammation and neurological deficits [5, 53], suggesting a role for the P38 kinase in ICH-induced brain injury. Altogether, our results suggest that the P38-MAPK pathway could represent a molecular target for inhibition of sEH and thus mediate its anti-inflammatory properties.

A major finding in our study was that inhibiting sEH after ICH reduced proinflammatory microglia/macrophage activation, which supports the conclusion from a previous study that showed EET suppressed proinflammatory macrophage activation while promoting the alternative activation of restorative macrophages [21]. In models of brain damage, the proinflammatory phenotype microglia/macrophages contribute to tissue injury while the restorative phenotype promotes repair [29, 54, 55]. Recent studies also show that reduction of proinflammatory microglia/macrophage activation correlated with lesion volume reduction and improvement in neurologic deficits following ICH [10, 11]. Herein, we demonstrate that genetic deletion or pharmacological inhibition of sEH after ICH reduced the expression of proinflammatory microglia/macrophage activation markers (IL-1β, IL-6, MIP-2, and CD16/32) at 1 and 4 days post-ICH. Notably, this reduced activation profile in AUDA-treated ICH mice was associated with improved long-term functional recovery and reduced brain tissue loss at 28 days post-ICH. These data implies that inhibiting sEH after TBI reduces proinflammatory microglia/macrophage activation and provides neuroprotection. However, previous studies have shown that microglia/macrophages become polarized toward proinflammatory or restorative phenotypes at different stages after ICH [11, 29]. Whether blocking the sEH activity affects the balance of microglia/macrophage polarization deserves further study.

We showed that both gene deletion and pharmacological inhibition of sEH reduced BBB disruption but did not alter hemorrhage volumes. Following ICH, both blood components (e.g., thrombin, hemoglobin, iron) and the inflammatory responses they induce contribute to BBB disruption [35]. We demonstrated that deletion and inhibition of sEH reduced MMP-9 activity, microglial/macrophage activation, and expression of pro-inflammatory molecules and attenuated neutrophil infiltration into the brain. Thus, inhibition of sEH probably protected BBB integrity through attenuation of the inflammatory responses rather through influencing hemorrhage volumes.

We observed that sEH was expressed in astrocytes and neurons, in addition to microglia/macrophages. Thus, apart from these anti-inflammatory actions, inhibition of the sEH activity may provide neuroprotection via other mechanisms. For example, a recent study reported that addition of 14,15-EET to astrocytes after oxygen-glucose deprivation (OGD) increased brain-derived neurotrophic factor (BDNF) expression and astrocyte survival. Additionally, administration of sEH inhibitors to cultured astrocytes after OGD increased vascular endothelial growth factor (VEGF) secretion, which then enhanced Akt pro-survival signaling in neurons leading to less neuronal cell death. Thus, inhibition of sEH may promote production of BDNF and VEGF from astrocytes to improve neuronal survival. Furthermore, inhibition of sEH exhibits direct neuroprotective properties, as evidenced by a report showing that pharmacological inhibition of sEH reduced cell death in cortical neurons exposed to hypoxic injury [56]. Closely related, overexpression of WT sEH exacerbated OGD-induced neuronal death in primary cortical neurons, which was reversed by addition of exogenous 14,15-EET [57]. Inhibition or deletion of sEH also provides protection against brain damage via a vascular mechanism. In experimental cerebral ischemia, gene deletion of sEH enhanced regional cerebral blood flow [16] and pharmacological inhibition of sEH improved cerebrovascular structure and microvascular density [15]. Future studies evaluating the effect of sEH inhibition/deletion on neuronal survival, astrocyte release of neurotrophic factors, and alterations in cerebral blood flow accompanying ICH-induced brain damage will elucidate the underlying effector mechanisms involved in its pathogenesis. Additionally, although we employed the most commonly used ICH model which most accurately mimics the spontaneous intracerebral bleeding and evolving hematoma expansion observed in patients, collagenase may induce an exaggerated inflammatory response. Thus, further experiments using different ICH models are necessary prior to clinical translation of these data.

## Conclusion

**Fig. 8:**
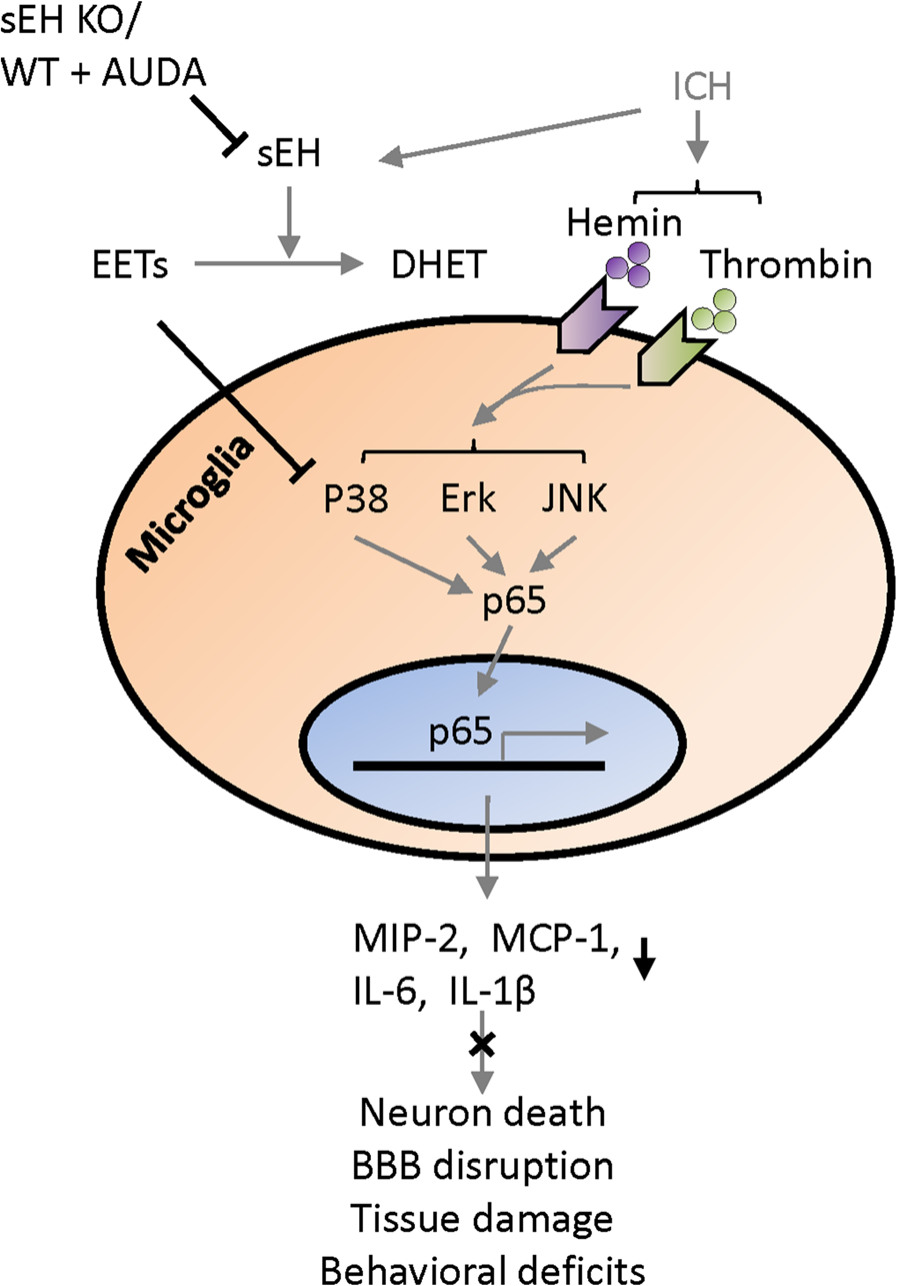
The schematic diagram indicates the potential mechanisms involved in sEH deletion or inhibition-induced protection in ICH mice. KO knockout, WT wild type, BBB blood-brain barrier

In conclusion, we show that both genetic deletion and pharmacological inhibition of sEH significantly reduced inflammatory responses, BBB permeability, and limited neurodegeneration after ICH (Fig. 8). Inhibition of sEH also improved long-term functional recovery, reduced chronic brain tissue loss, and suppressed proinflammatory microglia/macrophage activation. In cultured microglia, inhibition of sEH reduced activation of P38 MAPK and NF-κB signaling. Furthermore, the sEH inhibitor AUDA attenuated neurotoxicity induced by microglial conditioned media in vitro. These findings imply that sEH may provide a potential therapeutic target for secondary brain injury after ICH.

## Additional files

Additional file 1: Figure S1. Isolation of high purity microglia as verified by immunohistochemical analysis using microglia (Iba1, green) and astrocyte (GFAP, red) specific markers. Staining for nuclei of cells in culture by DAPI (blue) is also shown. The scale bar is 100 μm. (TIFF 1214 kb)

Additional file 2: Figure S2. Effects of saline and heat-inactivated collagenase on apoptosis and inflammatory responses in ipsilateral hemispheres 1 day after injection. Representative immunoblots and bar graphs show that there were no significant differences in (A) body weight change and protein levels of (B) cCP-3, (C) Iba1, and (D) IL-1β between the saline- and heat-inactivated collagenase-injected (HIC) mice. (TIFF 529 kb) (TIFF 601 kb)

Additional file 3: Figure S3. Bar graphs of (A) IL-1β, IL-6, and MIP-2 protein levels and (B) EET protein level, EET/14,15-DHET ratio, and 14,15-DHET protein level at 1 day post-ICH. Values are mean ± S.E.M.; ^##^ *P* < 0.01 and ^###^ *P* < 0.001 vs. sham group; *P < 0.05 and **P < 0.01 vs. vehicle group; ^†^ *P* < 0.05 vs. 1 μM AUDA group (*n* = 5–7 mice/group for cytokine ELISA and *n* = 4–5 mice/group for EET and 14,15-DHET ELISA, one-way ANOVA). (TIFF 529 kb)

Additional file 4: Figure S4. Effects of 10 μM AUDA on N2A cell viability as assessed by the MTT assay after exposure to thrombin or hemin for 24 h. Values are mean ± S.E.M.; ^###^ *P* < 0.001 vs. control group; **P < 0.01 vs. hemin-stimulation group (*n* = 4 experiments/group, one-way ANOVA). (TIFF 521 kb)

## Abbreviations

ANOVA: Analysis of variance
AUDA: 12-(3-Adamantan-1-yl-ureido)-dodecanoic acid
BBB: Blood-brain barrier
BDNF: Brain-derived neurotrophic factor
cCP-3: Cleaved caspase-3
COX-2: Cyclooxygenase-2
CYP450: Cytochrome P450
DHETs: Dihydroxyeicosatrienoic acids
DMEM: Dulbecco’s modified Eagle’s media
DMSO: Dimethyl sulfoxide
EETs: Epoxyeicosatrienoic acids
EEZE: 14,15-Epoxyeicosa-5(Z)-enoic acid
ELISA: Enzyme-linked immunosorbent assay
ERK: Extracellular signal-regulated kinases
FBS: Fetal bovine serum
FJB: Fluoro-Jade B
GFAP: Glial fibrillary acidic protein
Iba1: Ionized calcium-binding adaptor molecule 1
i.c.v.: Intracerebroventricular
ICH: Intracerebral hemorrhage
IL: Interleukin
iNOS: Inducible nitric oxide synthase
JNK: c-Jun N-terminal kinases
KO: Knockout
LPS: Lipopolysaccharide
MAPK: Mitogen-activated protein kinase
MCP-1: Monocyte chemoattractant protein-1
MIP-2: Macrophage inflammatory protein-2
MMP: Matrix metalloproteinase
mNSS: Modified neurological severity score
MPO: Myeloperoxidase
MTT: 3-(4,5-Dimethyl-2-thiazolyl)-2,5-diphenyl-2*H*-tetrazolium bromide
N2A: Neuro-2A
NeuN: Neuronal nuclei antigen
NO: Nitric oxide
OGD: Oxygen-glucose deprivation
P7: Postnatal day 7
PBS: Phosphate-buffered saline
ROS: Reactive oxygen species
RPMI: Roswell Park Memorial Institute
S.E.M.: Standard error of the mean
sEH: Soluble epoxide hydrolase
SP: Specificity protein
TdT: Terminal deoxynucleotidyl transferase
TNF: Tumor necrosis factor
TUNEL: Terminal deoxynucleotidyl transferase-mediated dUTP-biotin nick end labeling
VEGF: Vascular endothelial growth factor
WT: Wild type
