# Supplementary figures and images for: Genetic deletion or pharmacological inhibition of soluble epoxide hydrolase reduces brain damage and attenuates neuroinflammation after intracerebral hemorrhage
Source: J Neuroinflammation. 2017 Nov 25;14:230. doi: 10.1186/s12974-017-1005-4 (PMC5702198; doi:10.1186/s12974-017-1005-4)

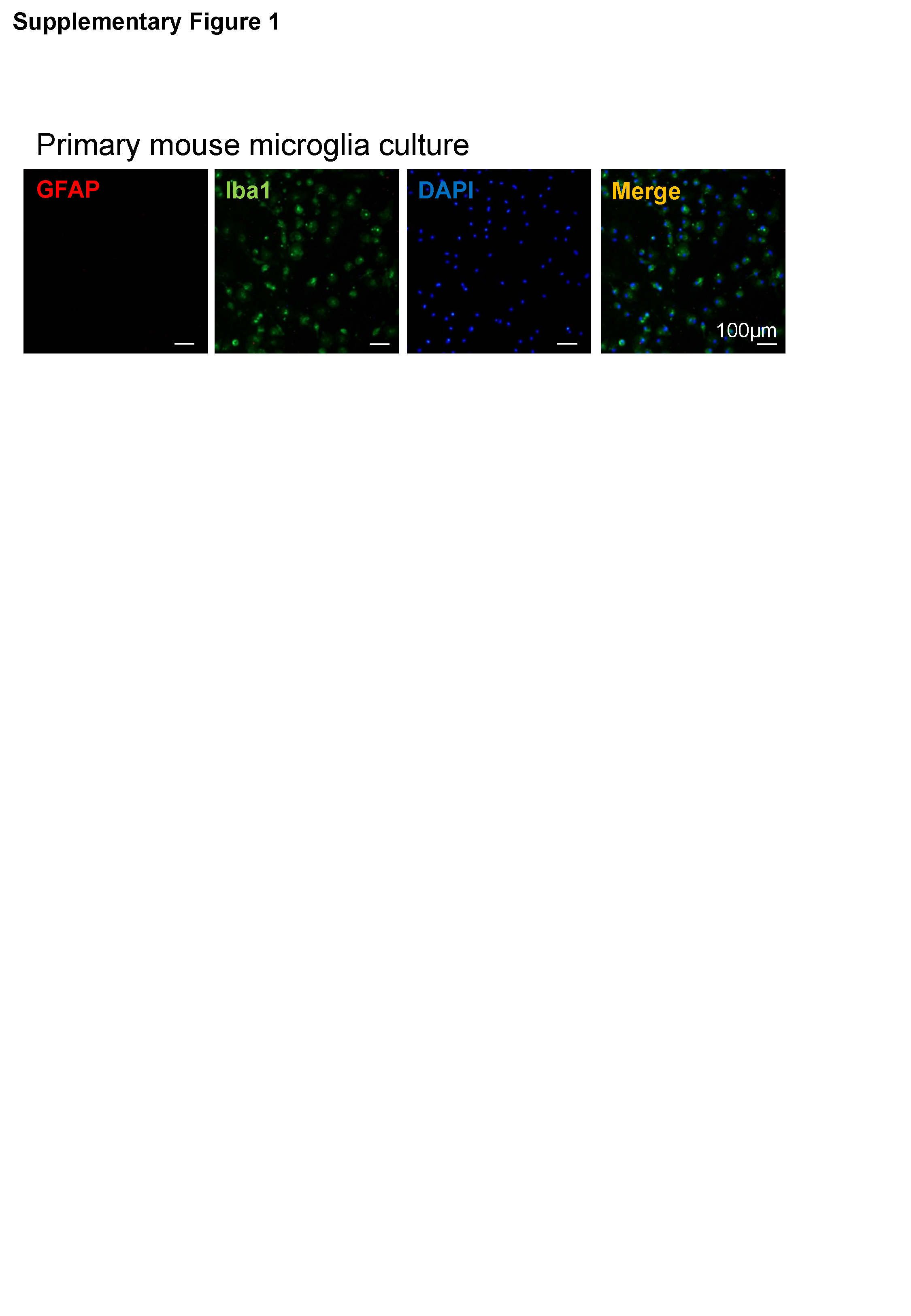

Supplement: Supplementary file 1 — Isolation of high purity microglia as verified by immunohistochemical analysis using microglia (Iba1, green) and astrocyte (GFAP, red) specific markers. Staining for nuclei of cells in culture by DAPI (blue) is also shown. The scale bar is 100 μm. (TIFF 1214 kb) [file 12974_2017_1005_MOESM1_ESM.tiff]

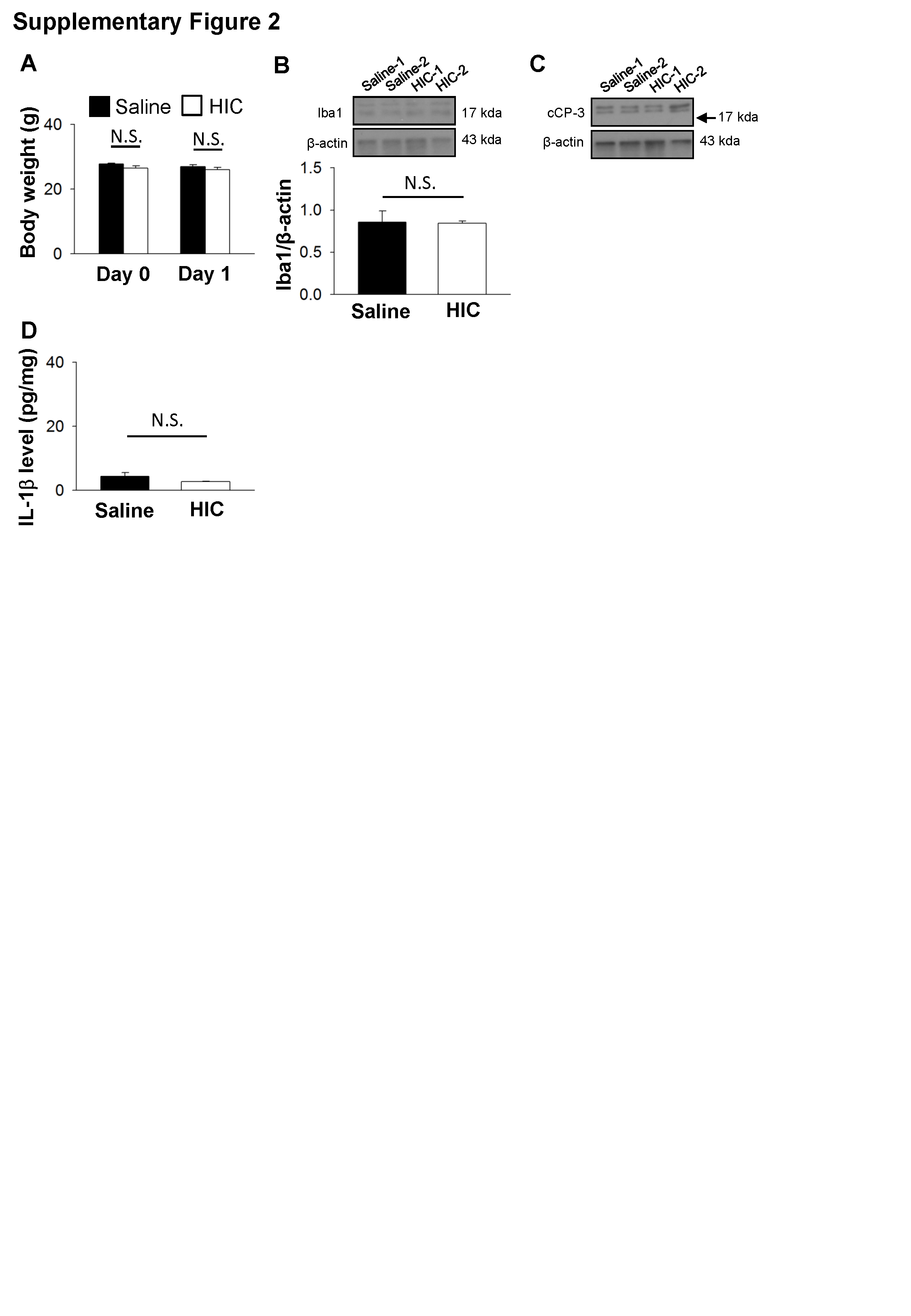

Supplement: Supplementary file 2 — Effects of saline and heat-inactivated collagenase on apoptosis and inflammatory responses in ipsilateral hemispheres 1 day after injection. Representative immunoblots and bar graphs show that there were no significant differences in (A) body weight change and protein levels of (B) cCP-3, (C) Iba1, and (D) IL-1β between the saline- and heat-inactivated collagenase-injected (HIC) mice. (TIFF 529 kb) (TIFF 601 kb) [file 12974_2017_1005_MOESM2_ESM.tif]

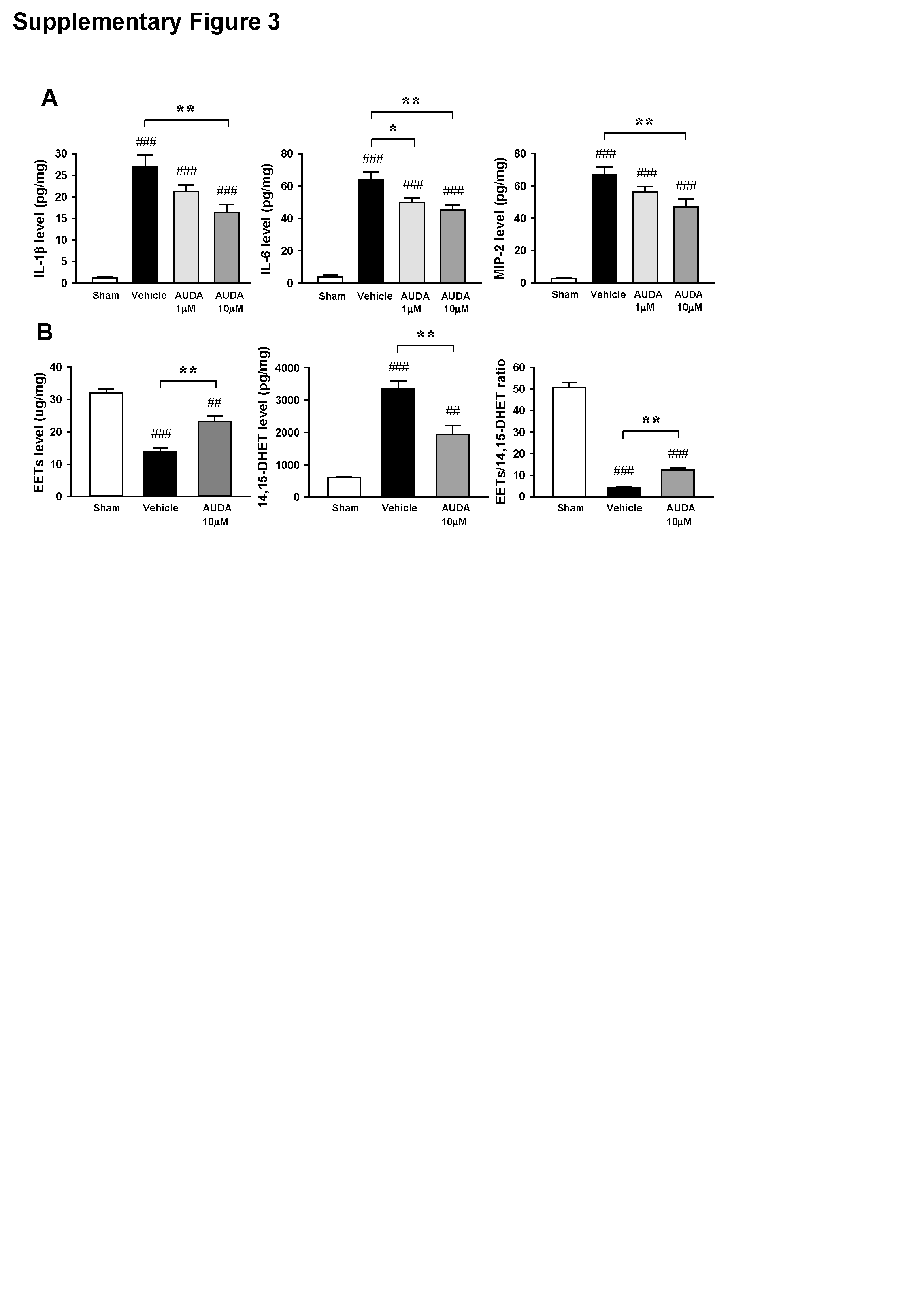

Supplement: Supplementary file 3 — Bar graphs of (A) IL-1β, IL-6, and MIP-2 protein levels and (B) EET protein level, EET/14,15-DHET ratio, and 14,15-DHET protein level at 1 day post-ICH. Values are mean ± S.E.M.; ## P < 0.01 and ### P < 0.001 vs. sham group; *P < 0.05 and **P < 0.01 vs. vehicle group; † P < 0.05 vs. 1 μM AUDA group (n = 5–7 mice/group for cytokine ELISA and n = 4–5 mice/group for EET and 14,15-DHET ELISA, one-way ANOVA). (TIFF 529 kb) [file 12974_2017_1005_MOESM3_ESM.tiff]

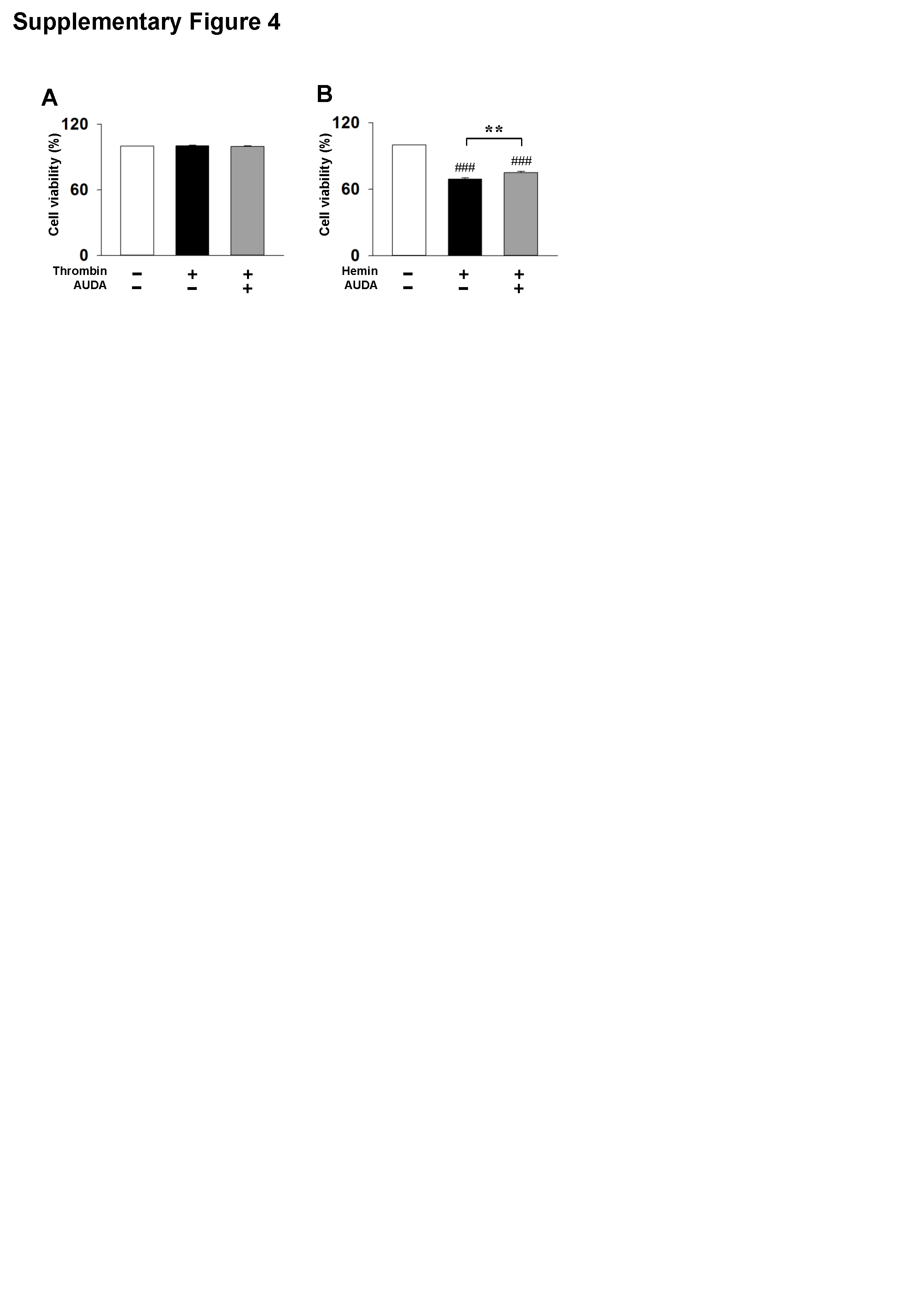

Supplement: Supplementary file 4 — Effects of 10 μM AUDA on N2A cell viability as assessed by the MTT assay after exposure to thrombin or hemin for 24 h. Values are mean ± S.E.M.; ### P < 0.001 vs. control group; **P < 0.01 vs. hemin-stimulation group (n = 4 experiments/group, one-way ANOVA). (TIFF 521 kb) [file 12974_2017_1005_MOESM4_ESM.tiff]
